# Chemical genetic analysis of enoxolone inhibition of *Clostridioides difficile* toxin production reveals adenine deaminase and ATP synthase as antivirulence targets

**DOI:** 10.1016/j.jbc.2024.107839

**Published:** 2024-09-27

**Authors:** Ravi K.R. Marreddy, Gregory A. Phelps, Kelly Churion, Jonathan Picker, Reid Powell, Philip T. Cherian, John J. Bowling, Clifford C. Stephan, Richard E. Lee, Julian G. Hurdle

**Affiliations:** 1 Center for Infectious and Inflammatory Diseases, Institute of Biosciences and Technology, Texas A&M Health Science Center, Houston, Texas, USA; 2 Department of Chemical Biology and Therapeutics, St Jude Children’s Research Hospital, Memphis, Tennessee, USA; 3 Graduate School of Biomedical Sciences, St Jude Children’s Research Hospital, Memphis, Tennessee, USA; 4 Center for Translational Cancer Research, Institute of Biosciences and Technology, Texas A&M Health Science Center, Houston, Texas, USA

**Keywords:** toxins, sporulation, adenine deaminase, ATP synthase, purine metabolism, phosphate metabolism

## Abstract

Toxins TcdA and TcdB are the main virulence factors of *Clostridioides difficile*, a leading cause of hospital-acquired diarrhea. Despite their importance, there is a significant knowledge gap of druggable targets for inhibiting toxin production. To address this, we screened nonantibiotic phytochemicals to identify potential chemical genetic probes to discover antivirulence drug targets. This led to the identification of 18*β*-glycyrrhetinic acid (enoxolone), a licorice metabolite, as an inhibitor of TcdA and TcdB biosynthesis. Using affinity-based proteomics, potential targets were identified as ATP synthase subunit alpha (AtpA) and adenine deaminase (Ade, which catalyzes conversion of adenine to hypoxanthine in the purine salvage pathway). To validate these targets, a multifaceted approach was adopted. Gene silencing of *ade* and *atpA* inhibited toxin biosynthesis, while surface plasmon resonance and isothermal titration calorimetry molecular interaction analyses revealed direct binding of enoxolone to Ade. Metabolomics demonstrated enoxolone induced the accumulation of adenosine, while depleting hypoxanthine and ATP in *C. difficile*. Transcriptomics further revealed enoxolone dysregulated phosphate uptake genes, which correlated with reduced cellular phosphate levels. These findings suggest that enoxolone's cellular action is multitargeted. Accordingly, supplementation with both hypoxanthine and triethyl phosphate, a phosphate source, was required to fully restore toxin production in the presence of enoxolone. In conclusion, through the characterization of enoxolone, we identified promising antivirulence targets that interfere with nucleotide salvage and ATP synthesis, which may also block toxin biosynthesis.

*Clostridioides difficile* infection (CDI) is a leading cause of hospital-acquired diarrhea, in 2017 causing 20,500 in-hospital deaths in the United States from 462,100 cases (1). Broad-spectrum antibiotics are the main risk factor for CDI, as they promote dysbiosis and *C. difficile* colonization (2). Metronidazole and vancomycin have been the main antibiotic treatments for CDI, but 20% or more patients experience recurrence following therapy (3). These two antibiotics also inhibit the growth of important gut flora and therefore further perturb CDI-associated dysbiosis (4, 5). Therefore, a medical need exists for novel therapeutics that is more targeted to *C. difficile* pathophysiology.

*C. difficile* produces two glycosyltransferase toxins (TcdA and TcdB), which are responsible for CDI symptoms (6). Consequently, substantial research efforts has been devoted to developing therapeutics that inactivate *C. difficile* toxins (7, 8, 9), with bezlotoxumab, an Food and Drug Administration-approved monoclonal antibody to TcdB, being the leading example (8). Conversely, there is a paucity of discovery-oriented research to identify inhibitors that block the cellular synthesis of TcdA and TcdB. The biosynthesis of TcdA and TcdB is regulated by various sigma factors, environmental, and nutritional changes (6). For example, glucose impedes toxin production through catabolite repression, whereby the catabolite control protein A (CcpA) binds to the promoter of the alternative sigma factor *tcdR*, whose gene product enhances *tcdA* and *tcdB* transcription (10, 11, 12); during nutrient excess and high intracellular concentrations of GTP, the nutritional regulator CodY binds to the *tcdR* promoter to repress *tcdA* and *tcdB* transcription (13); and lastly, the activation of Stickland metabolism of proline is thought to suppress toxin biosynthesis (14). Yet, there have been limited systematic efforts to discover inhibitors that either target these known pathways or others affecting toxin production (15).

To identify inhibitors of toxin biosynthesis, we focused on a small panel of naturally occurring nonantibiotics from different phytochemicals classes (Table S1). The potential for medicinal and food-based phytochemicals as treatments for CDI is underscored by berberine, which has weak antibacterial activity and is efficacious in mice with CDI (16); however, berberine’s mode of action against *C. difficile* is unclear. The fact that plant-derived compounds often display weak antibacterial activities (17, 18) imply that they could be promising chemical starting points for nonantibiotics that inhibit TcdA and TcdB biosynthesis. We therefore explored different classes of phytochemicals to discover that the pentacyclic triterpenoid 18*β*-glycyrrhetinic acid (enoxolone) blocks cellular production of TcdA and TcdB. Subsequent use of enoxolone as a chemical genetic probe identified that it inhibited toxin production through a complex mode of action involving disruptions to cellular purine, ATP and phosphate metabolisms.

## Results

### Identification of *C. difficile* toxin biosynthesis inhibitors

We assembled a small panel (n = 57) of phytochemicals/metabolites from different structural classes to identify inhibitors of *C. difficile* toxin production that did not substantially prevent bacterial growth. The panel included phytochemicals with known medicinal properties, as described in Table S1. *C. difficile* R20291 was selected as the test strain, as it is a representative of hypervirulent epidemic ribotype 027 (19). To identify inhibitors, we tested molecules at 100 μM and triaged those inhibiting growth by ≥50%. After triaging growth inhibitors, toxins were quantified in the cultures that were exposed to the remaining compounds. This was done using a cytopathic assay that was based on TcdB-induced rounding of Vero cells (20) and quantifying toxins from automated morphometric analysis of cell shape and surface area covered in 384 well plates. Similar automated assays have been reported for *C. difficile* TcdB (21). Presumptive compound hits were then confirmed by ELISA to quantify the amounts of TcdA and TcdB in the culture supernatants.

**Figure 1:**
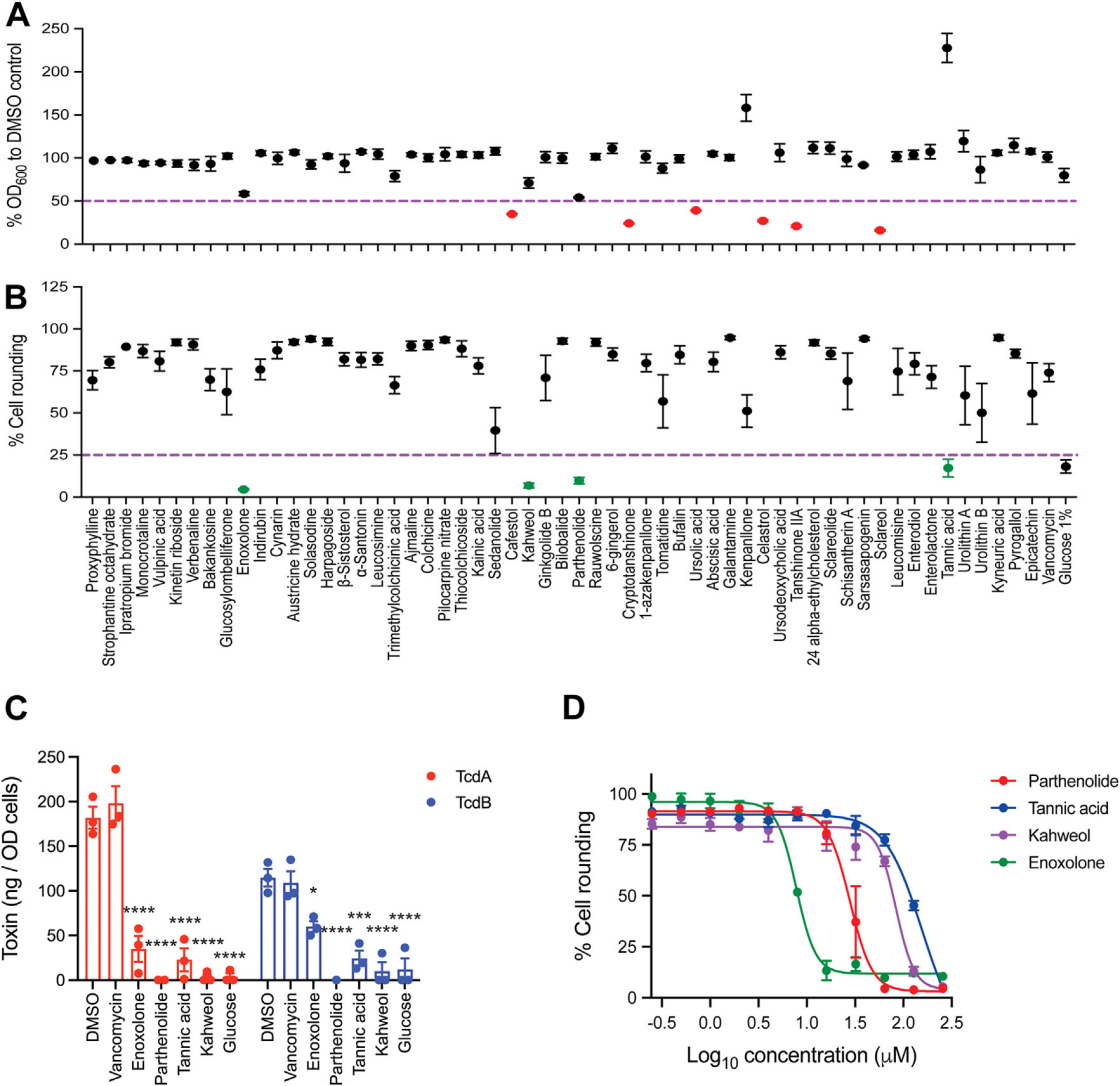
**Screening for inhibitors of *Clostridioides difficile* toxin biosynthesis.** R20291 in BHI in 96 well plates was exposed to 100 μM of phytochemicals for 24 h to measure: (*A*) effects on growth (*A*_600_ nm); growth inhibitors are indicated in *red* and were triaged by comparison with the DMSO control (n = 3 biological replicates). *B*, toxins were quantified by cytopathic cell rounding; inhibitors of toxin synthesis are indicated in *green* (n = 4 biological replicates). Controls were vancomycin (0.5 μM) and glucose 1% (w/v). The *purple line* indicates cutoff criteria for compound selection. *C*, toxins (TcdA and TcdB) in culture supernatants were quantified by ELISA; enoxolone (100 μM) inhibited synthesis of TcdA by 81.18 ± 12.68% and TcdB by 47.40 ± 9.02% (n = 3 biological replicates; statistical significance was assessed by one-way ANOVA with Tukey’s test; ∗*p* < 0.05, ∗∗∗*p* < 0.001 and ∗∗∗∗*p* < 0.0001 in Graphpad prism 9.3.1). *D*, cell rounding assay showing dose-response curves of toxin biosynthesis inhibitors parthenolide, tannic acid, kahweol, and enoxolone (n = 4 biological replicates). Respectively, EC_50_s and Hill slopes were 27.93 μM and −3.75; >80 μM and −2.24; >80 μM and −4.56; and 7.77 μM and −4.33; the negative Hill slopes are indicative of downhill inhibition curves, where increases in drug concentration caused a decrease in cell rounding, with narrow thresholds between effective and ineffective drug concentrations; this suggests the compounds have complex modes of actions. Toxins, namely TcdB, were quantified by cell rounding against Vero cells and used for biological replicates. Data in all plots are shown as mean ± SEM. BHI, brain heart infusion; DMSO, dimethyl sulfoxide.

Results showed that of the 57 compounds, six were eliminated as they inhibited growth by >50% (Fig. 1*A*). From the remaining 51 compounds, four hits reduced the cytopathic effects of *C. difficile* on Vero cells, as follows: enoxolone (15.02 ± 0.87%), parthenolide (16.21 ± 1.15%), kahweol (20.74 ± 8.55%), and tannic acid (14.57 ± 2.71%). In comparison, dimethyl sulfoxide (DMSO) treated *C. difficile* caused 90.86 ± 0.69% of Vero cell rounding, whereas the positive-control glucose (1% w/v) caused 20.31 ± 4.89% of cell rounding (Fig. 1*B*). ELISA validated that there was reduced toxins in culture supernatants for TcdA (81.18–99.99% reduction) and TcdB (47.4–91.2% reduction) (Fig. 1*C*). With glucose, percentage inhibition of TcdA and TcdB were 98.14 ± 3.11% and 90.79 ± 15.83, respectively; vancomycin did not inhibit toxin production at a growth permissive concentration (0.5 μM). In our test conditions, the ratio of TcdA to TcdB in culture supernatants was 1.8 to 1, which is consistent with reports that *tcdA* is more highly expressed than *tcdB* (∼2:1 ratio) and *C. difficile* produces more TcdA than TcdB (about 2:1–3:1 ratio) (6, 12).

### Prioritization of enoxolone and effects on toxin synthesis in varying *C. difficile* strains

Assessment of dose-responses using the cell rounding assay showed that kahweol and tannic acid had high half maximal effective concentration (EC_50_) values of greater than 80 μM, when compared to parthenolide (EC_50_ = 27.93 μM) and enoxolone (7.77 μM) (Fig. 1*D*). This suggested that enoxolone and parthenolide inhibit *C. difficile* toxin production. However, enoxolone was prioritized for further studies due its greater potency, chemical tractability for designing molecular probes, and previous reports of related pentacyclic triterpenoids showing antivirulence properties (22). Enoxolone is a pentacyclic triterpenoid aglycone metabolite of glycyrrhizin, the main bioactive component of licorice root extracts (*Glycyrrhiza* [sweet root]) (23). It exhibits a range of pharmacological properties including antitumor, antiinflammatory, and antiviral activities (23). Interestingly, glycyrrhizin did not inhibit toxin production (Fig. S1), suggesting that its glucuronic acid disaccharide moiety hindered cellular activity.

**Figure 2:**
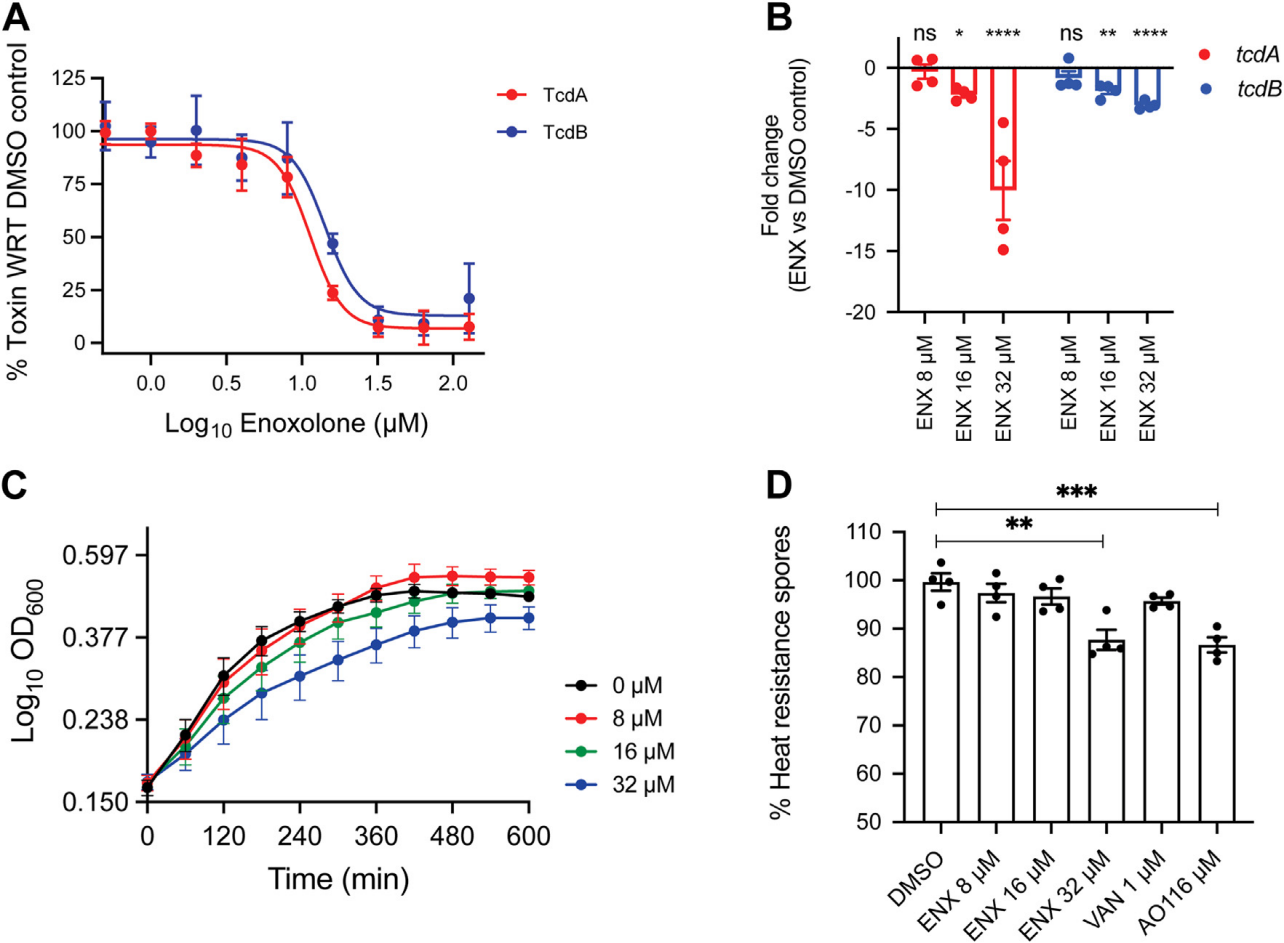
**Characterization of enoxolone (ENX) activity against *Clostridioides difficile*.***A*, dose response of ENX against R20291. Exponential cultures (*A*_600_ nm ≈ 0.3) were exposed to DMSO or 2 fold increasing concentrations of ENX (n = 3 biological replicates). After 24 h, TcdA and TcdB were quantified by ELISA. Respectively, EC_50_s and Hill slopes were for TcdA (11.38 mM and −4.04) and TcdB (14.29 and −3.96), which are indicative of downhill inhibition curves, where increasing drug concentration significantly decreases toxin production about a narrow threshold between effective and ineffective drug concentrations; this suggests a complex mode of action. *B*, effect of ENX on mRNA levels of *tcdA* and *tcdB*, as determined by RT-qPCR. Cultures (*A*_600_ ≈ 0.3; n = 4 biological replicates) were exposed to ENX, and mRNA analyzed after 9 h; the fold change was calculated relative to the DMSO control. Statistical significance was assessed from ΔCt values, comparing DMSO and the different ENX treated samples by two-way ANOVA with Tukey’s test: ∗*p* < 0.05, ∗∗*p* < 0.01 and ∗∗∗∗*p* < 0.0001 in Graphpad prism 9.3.1. *C*, effect of ENX on growth of R20291. Exponential cells (*A*_600_ nm ≈ 0.2; n = 4 biological replicates) were treated with DMSO or ENX at ½×, 11× and 22× EC_50_ of 8 μM. *D*, effect of ENX on sporulation. Exponential cells (*A*_600_ nm ≈ 0.3) were exposed to compound or DMSO for 5 days; total viable counts and spores were then enumerated; ENX was used at ½x, 11× and 22× EC_50_ of 8 μM. Controls were vancomycin (VAN; 1 μM) and acridine orange (AO = 116 μM); n = 4 biological replicates are shown as mean ± SEM and statistical significance was by one-way ANOVA with Tukey’s test: ∗∗*p* < 0.01 and ∗∗∗*p* < 0.001 in Graphpad prism 9.3.1. DMSO, dimethyl sulfoxide.

Dose-dependent effects of enoxolone on toxin production by various ribotype 027 strains were further evaluated by ELISA and cell rounding assays. The EC_50_s for inhibition of TcdA and TcdB production were similar to the EC_50_s seen in the cell rounding assay (Figs. 2*A* and S2, *A* and *B*), indicating that enoxolone similarly affected both toxins and that 16 μM was an effective concentration against ribotype 027. Against other major CDI-associated ribotypes (PCR ribotypes 014, 020, 078, and 106) (1) enoxolone at 32 μM (*i.e.*, 22× the adopted EC_50_ of 16 μM) reduced production of TcdA by ≥59.0% and TcdB by ≥77.6% (Fig. S2, *C* and *D*).

### Effect of enoxolone on transcription of toxin genes

We exposed cells to enoxolone for 9 h and recovered stationary phase cells to measure effects on *tcdA* and *tcdB* transcription. This revealed that enoxolone reduced *tcdA* and *tcdB* transcripts in a dose-dependent manner, as 8 μM (*i.e.*, 0.5× EC_50_ for toxin biosynthesis inhibition) did not have a significant effect, whereas 16 μM and 32 μM (*i.e.*, 11× and 22× EC_50_, respectively) reduced transcription as follows: (*tcdA* [2.17 ± 0.49 and 10.1 ± 4.2–fold]) and *tcdB* ([1.8 ± 0.3 and 2.9 ± 0.36–fold]), respectively (Fig. 2*B*). The greater impact on *tcdA* expression at 32 μM might be due to *tcdA* being more highly expressed (12), which might provide a greater measurable margin for changes in transcript amounts. Since *tcdA* is more highly expressed than *tcdB* (12) and both toxins are significantly affected by enoxolone (Figs. 2*A* and S2, *A*–*D*), we adopted TcdA as a general readout for inhibition of toxin production in most subsequent experiments.

### Effect of enoxolone on growth and sporulation

Enoxolone only modestly affected growth of R20291 at the EC_50_ (16 μM) for inhibition of toxin production (Fig. 2*C*), but it delayed growth at a supra concentration of 32 μM (Fig. 2*C*). Because regulatory mechanisms for toxin production and sporulation can intersect in *C. difficile* (6), we tested the effect of enoxolone on sporulation. Cultures were treated with compounds for 5 days before measuring total viable counts and spores (24). This revealed enoxolone inhibited sporulation at 32 μM (Figs. 2*D* and S2*E*), which was comparable to the positive control acridine orange (116 μM) that is known to inhibit sporulation without affecting growth (25). In contrast, the comparator vancomycin (at 11 or 2× its MIC of 1 μM), did not inhibit sporulation (Figs. 2*D* and S2*E*).

### Exploration of cellular targets of enoxolone

To identify and validate cellular targets of enoxolone, we used a combination of chemical and molecular genetics techniques, alongside a review of known pathways affecting *C. difficile* toxin synthesis (6, 26). Firstly, we adopted activity-based proteomic profiling (Fig. S3*A*) using a synthetic alkyne derivative of enoxolone (compound 3511) (Fig. S3*B*). The probe inhibited toxin biosynthesis in R20291 in a manner similar to enoxolone (Fig. S3*C*). For activity-based proteomic profiling, compound 3511 was immobilized to carboxymethylrhodamine (TAMRA) azide through CuAAC click reaction and then incubated with R20291 cell lysates. In parallel, a control mock reaction was done with unlabeled enoxolone. Visualization of click labeled proteins by in-gel fluorescence predominantly showed bands of ∼50 to ∼60 kDa (Fig. S3*D*), suggesting enoxolone bound to multiple targets. However, all proteins eluted from the streptavidin magnetic beads were subjected to MALDI-TOF mass spectrometry. This identified that there were 24 and 17 proteins in the click and mock reactions, respectively. Of these, 14 proteins were unique to the click labeled compound 3511 (Table S2 and Fig. S3*E*). To prioritize proteins for molecular genetic validation, we took a holistic approach that was guided by both the results of the in-gel fluorescence and existing knowledge of metabolic and regulatory networks affecting *C. difficile* toxin production (6, 26). Hence, seven proteins were not considered since their molecular weights were either <45 kDa or >70 kDa (outside of the molecular weight range for prominent bands seen from in-gel fluorescence) (Table S2).

Of the remaining seven proteins, we focused on NADP-dependent glyceraldehyde-3-phosphate dehydrogenase (GapN), ATP synthase subunit alpha (AtpA), and adenine deaminase (Ade). The activities of these proteins are predicted to affect metabolic networks that regulate toxin production as follows. GapN is part of the glycolytic pathway that catalyzes the breakdown of glucose (27). ATP synthase is thought to couple ATP synthesis from the sodium/proton gradient generated from the coupled oxidation of ferredoxin and reduction of NAD+ by the RNF complex (27). Ade catalyzes the early steps of the purine salvage pathway, in which it converts adenine to hypoxanthine that feeds into multiple pathways for purine biosynthesis (28); the salvage pathway is thought to be a more energy saving approach for purine biosynthesis than the *de novo* pathway (29).

**Figure 3:**
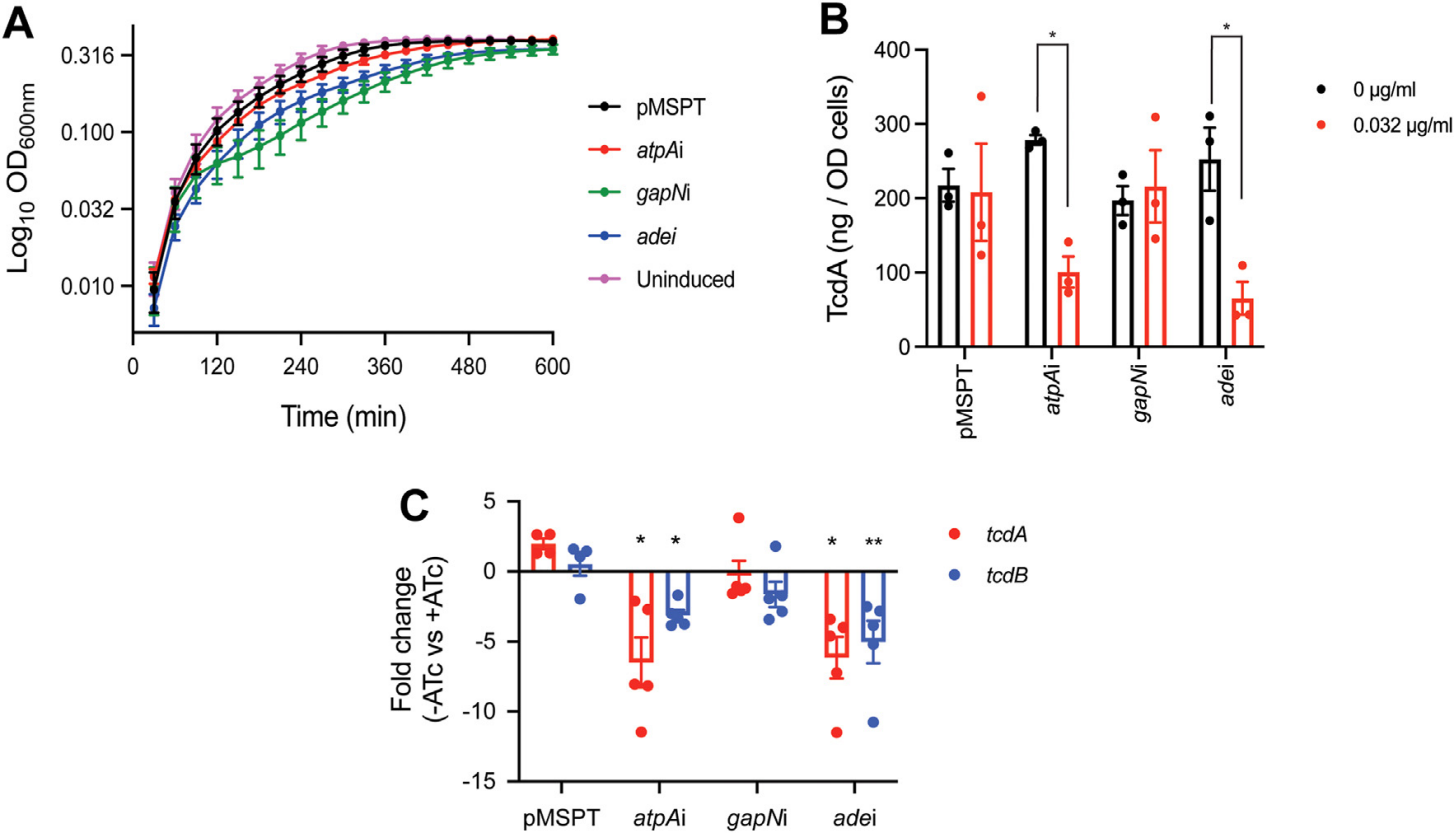
**Effect of silencing genes for which proteomics suggested to encode targets of enoxolone.** Analyzed were R20291 with the empty vector control (pMSPT) or R20291 with pMSPT expressing antisense RNA (asRNA) to *ade*, *gapN*, or *atpA*; 0.032 μg/ml of anhydrotetracycline (ATc) was used for induction. *A*, growth kinetics were analyzed for the strains *i.e.*, pMSPT (*black*), *atpAi* (*red*), *gapN*i (*green*), and *adei* (*blue*) in microdilution 96-well plate (n = 4 biological replicates). As a growth control, R20291 bearing pMSPT without ATc exposure was analyzed. *B*, TcdA was analyzed at 24 h after induction of asRNA. Data from three biological replicates are shown as mean ± SEM and analyzed by two-way ANOVA with Tukey’s test: ∗*p* < 0.05 in Graphpad prism 9.3.1. *C*, mRNA levels of toxin genes (*tcdA* and *tcdB*) were analyzed by reverse transcriptase quantitative PCR (n = 3 biological replicates) from exponential cultures (*A*_600_ nm ≈ 0.3) treated with 0 or 0.032 μg/ml of ATc for 6 h. The fold change was calculated for mRNA from drug free and ATc exposed cultures. Significance was analyzed by comparing the ΔCt values of samples with ATc at 0 μg/ml *versus* those with ATc 0.032 μg/ml by two-way ANOVA with Tukey’s test: ∗*p* < 0.05 and ∗∗*p* < 0.01 in Graphpad prism 9.3.1. Ade, adenine deaminase; atpA, ATP synthase subunit alpha; GapN, NADP-dependent glyceraldehyde-3-phosphate dehydrogenase.

To first examine *gapN*, *atpA*, and *ade* roles in toxin biosynthesis, we silenced these genes with antisense RNA (asRNA) nucleic acids and quantified toxin production and transcription of *tcdA* and *tcdB*. AsRNA fragments were expressed from the Ptet promoter in pMSPT and induced with a growth permissive concentration of 0.032 μg/ml anhydrotetracycline (ATc) (Fig. S4*A*) (24). Induction of asRNA for both *gapN* and *ade* caused moderate effects on growth while *atpA* showed no effects (Fig. 3*A*). Transcripts for *atpA*, *ade*, and *gapN* were reduced by ∼10 to 30–fold (Fig. S4*B*), but only *atpA* and *ade* silencing diminished TcdA production as well as the transcription of *tcdA* and *tcdB* (Figs. 3, *B* and *C*, and S4*C*). TcdA levels were reduced by 44.90 ± 17.35% and 68.71 ± 28.68% for *atpA* and *ade* silencing, respectively. Silencing of *atpA* and *ade*, but not *gapN*, synergized with enoxolone to inhibit toxin production, as determined using the cell rounding assay, and this occurred in an ATc concentration-dependent manner (Fig. S4*D*). We therefore further studied Ade and AtpA roles in toxin production.

### Enoxolone binds to adenine deaminase

**Figure 4:**
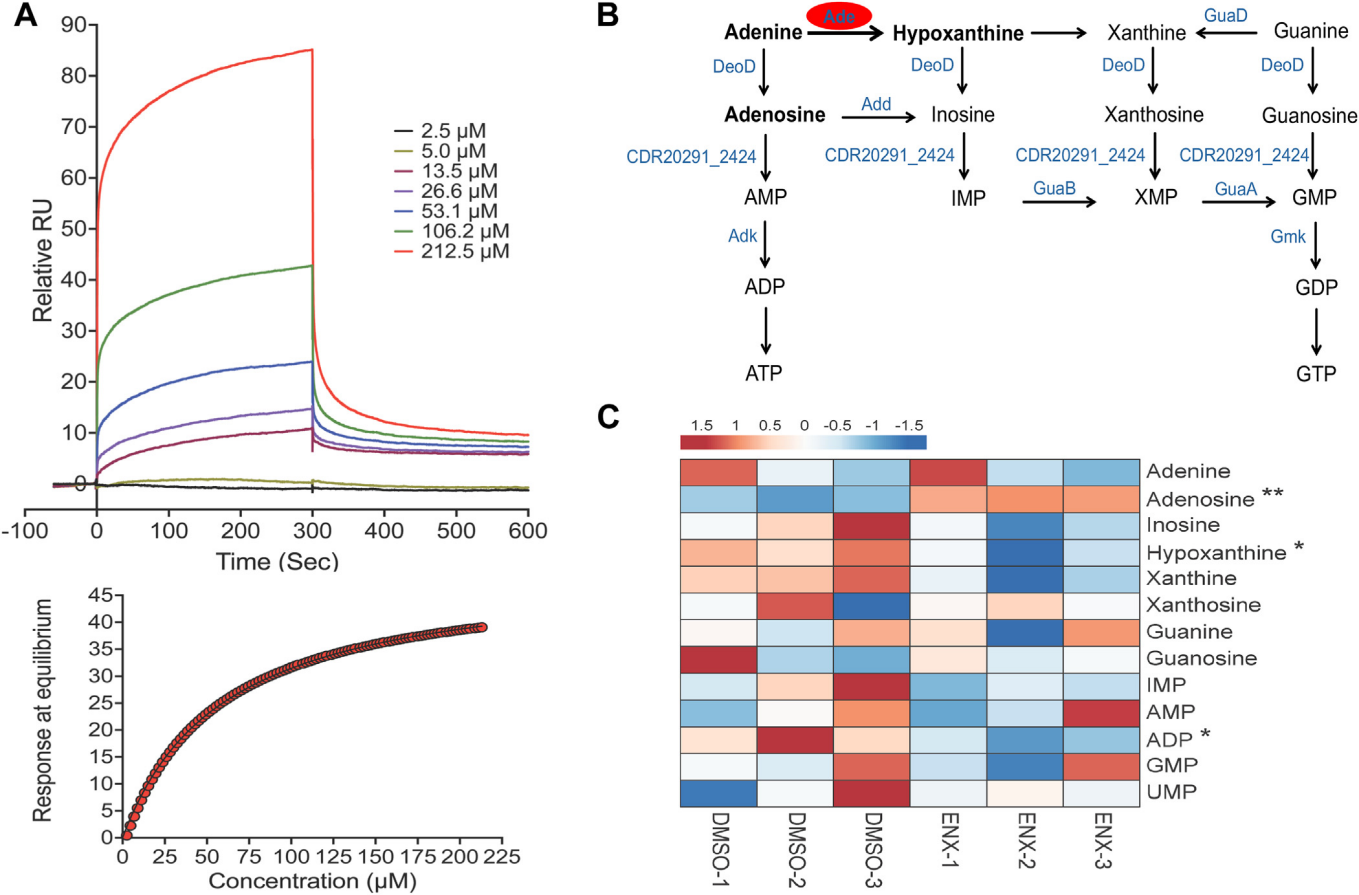
**Enoxolone (ENX) binds to adenine deaminase and inhibits purine metabolism.***A*, molecular interaction analysis using surface plasmon resonance (SPR). Dose response sensograms with different concentrations of ENX (*top panel*) shown as a representative of four replicates. The *bottom panel* shows the corresponding analysis of response at equilibrium of the experiment in the top panel (K_d_ = 52 μM for the replicate and 33.73 ± 14.14 μM overall). *B*, schematic representation of purine salvage pathway adapted from Kyoto Encyclopedia of Genes and Genomes (KEGG number T00998 for strain R20291), showing adenine deaminase’s role in converting adenine to hypoxanthine. Enzymes involved in this pathway are indicated in *blue* color. Ade = adenine deaminase; Add = adenosine deaminase; DeoD = purine nucleoside phosphorylase; CDR20291_2424 = putative membrane-associated 5′-nucleotidase/phosphoesterase; Adk = adenylate kinase; GuaD = guanine deaminase; GuaB = inosine-5′-monophosphate dehydrogenase; GuaA = GMP synthase [glutamine-hydrolyzing]; Gmk = guanylate kinase. *C*, LC-MS/MS analysis of purine metabolites. *C. difficile* R20291 cells (*A*_600_ ≈ 0.3) was exposed to ENX (16 μM) or 1% (v/v) DMSO for 3 h. Heatmaps generated with ClustVis software shows relative quantities of metabolites; *red* and *blue* color intensities indicate levels of metabolites that were increased or decreased, respectively. DMSO, dimethyl sulfoxide; LC-MS/MS, liquid chromatography with tandem mass spectrometry. Statistical significance was assessed by two-tailed unpaired t-tests; ∗*p* < 0.05, *p* < 0.01 in Graphpad prism 9.3.1.

Surface plasmon resonance (SPR) was conducted to measure the binding of enoxolone to Ade. The Ade protein with N-terminal His-tag was adhered to the SPR chip *via* a His-tag antibody and testing of enoxolone at 1.18 to 100 μg/ml (2.5–212.5 μM) indicated it bound to Ade in a concentration-dependent manner with a dissociation constant (K_d_) of 34.56 ± 13.43 μM (Fig. 4*A*). For these experiments, we adopted 6-chloropurine as a control since it was reported to inhibit *Escherichia coli* Ade, while several other adenine analogs (*e.g.*, 6-methylpurine) did not (28). However, against *C. difficile* Ade, 6-chloropurine was a weak binder with a K_d_ of 264 ± 42.9 μM (Fig. S5), while vancomycin (3.94 ± 1.52 M) had weak affinity, in the molar range. The binding of enoxolone to *C. difficile* Ade was confirmed by isothermal titration calorimetry (ITC), with K_d_ of 19.74 ± 9.74 μM (Fig. S5). Based on the ITC, enoxolone was predicted to make multiple binding interactions with Ade (mean N-value = 5.22), in contrast to the substrate adenine (mean N-value = 2.32); we speculate that enoxolone engages Ade in a manner that is dissimilar to the substrate adenine (Fig. S5).

### Effect of enoxolone on nucleotide metabolism

**Figure 5:**
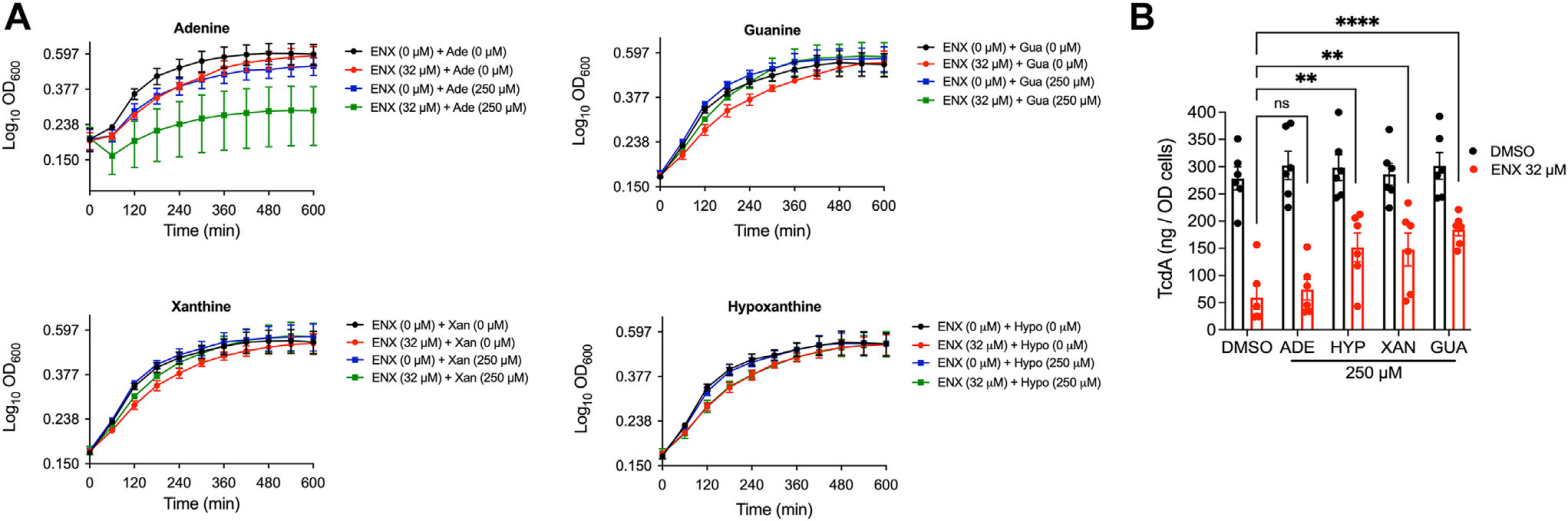
**Relationship between enoxolone (ENX) effects on growth, toxin production, and purine metabolism.***A*, growth kinetics of exponential R20291 (n = 3 biological replicates) in 1% (v/v) DMSO or ENX (32 μM) with or without various purine derivatives (250 μM). *B*, quantification of TcdA in R20291 cultures exposed to purine derivatives (250 μM) in presence of DMSO or ENX (32 μM); data from six biological replicates, shown as mean ± SEM, were statistically analyzed by two-way ANOVA with Tukey’s test ∗∗*p* < 0.01 and ∗∗∗*p* < 0.001 in Graphpad prism 9.3.1. DMSO, dimethyl sulfoxide.

Because Ade participates in purine metabolism (Fig. 4*B*), converting adenine to hypoxanthine, we adopted targeted metabolomics to analyze intracellular changes to nucleotide metabolites. When R20291 cells were exposed to 16 μM enoxolone there were significant differential increases and decreases in the intracellular concentrations of adenosine and hypoxanthine, respectively (Fig. 4*C*). Interestingly, ADP levels were significantly reduced in cells (Fig. 4*C*). These results suggested that enoxolone disrupted purine metabolism, likely causing intracellular depletion of hypoxanthine. We therefore tested whether supplementation with purines affected enoxolone effects on growth and toxin production. Growth was inhibited when cells were exposed to adenine (250 μM), and this was more pronounced in the presence of enoxolone (Fig. 5*A*). This suggests that intracellular accumulation of adenine is toxic to *C. difficile*. While hypoxanthine, xanthine, and guanine alone (at 250 μM) did not impact cell growth, only xanthine and guanine alleviated growth suppression by 32 μM enoxolone (Fig. 5*A*). Toxin production (*i.e.*, TcdA) was also partially restored by hypoxanthine, xanthine, and guanine by 2.55, 2.48, and 3.09-fold, respectively, when compared to cultures that were exposed to enoxolone (32 μM) without the metabolites (Fig. 5*B*); however, adenine had no effect. Taken together, these observations suggest adenine deaminase is blocked by enoxolone, which suppress the downstream cellular pools of hypoxanthine, xanthine, and guanine and their supplementation in media partially reverses enoxolone inhibition of toxin biosynthesis. The inability to fully restore toxin production also pointed to additional mechanisms.

### Decrease of cellular ATP by enoxolone

**Figure 6:**
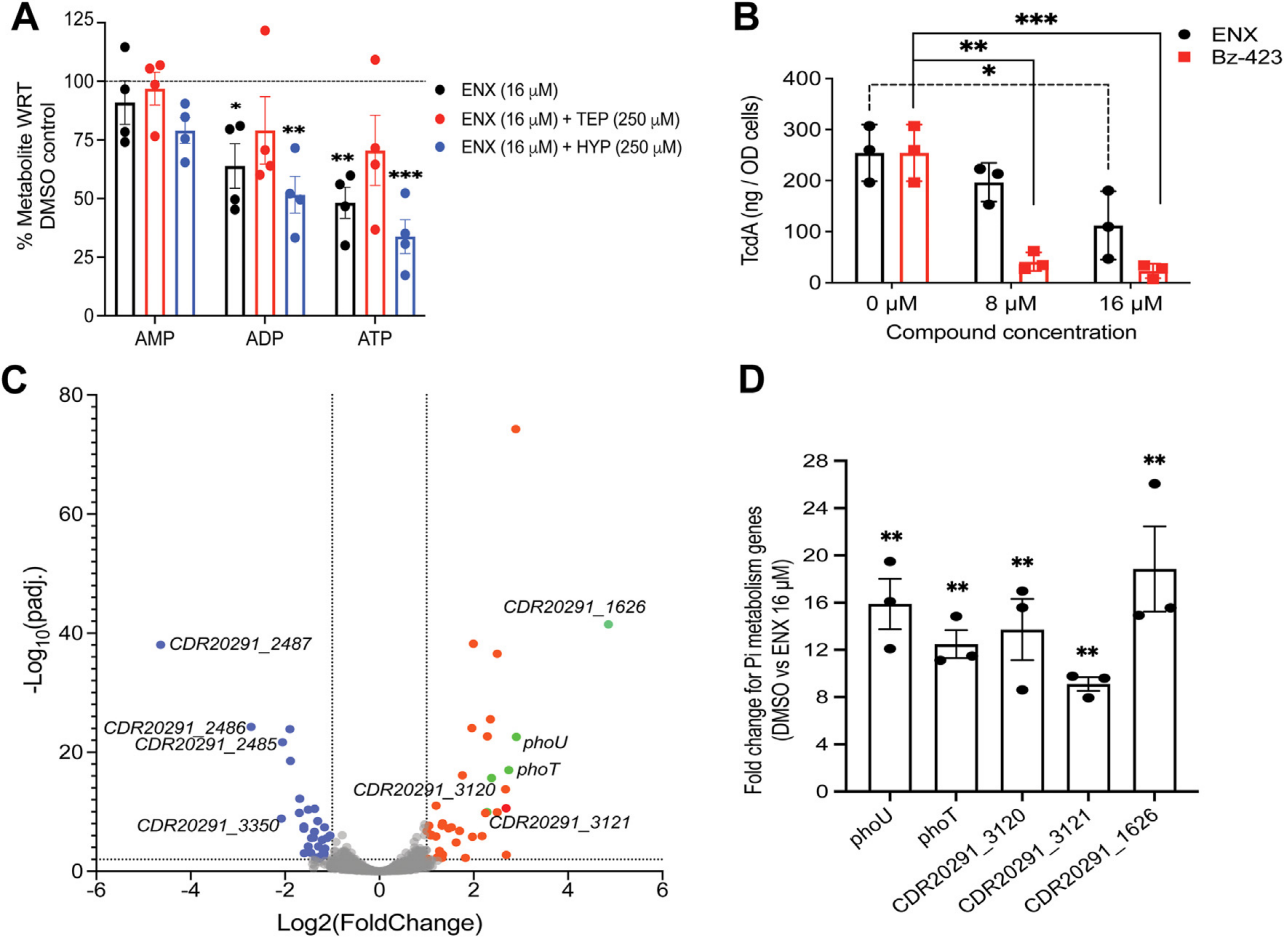
**Analysis of effects of enoxolone on cellular ATP, toxin production, and global transcriptome.***A*, quantification of intracellular pools of AMP, ADP, and ATP in *Clostridioides difficile* R20291 cells exposed to 16 μM of enoxolone. Cells in early exponential growth phase (*A*_600_ ≈ 0.3) were exposed to DMSO or enoxolone (ENX) in presence of 250 μM triethyl phosphate (*red bars*) or 250 μM hypoxanthine (*blue bars*). Cultures (n = 4 biological replicates) were harvested 3 h after exposure to ENX, and metabolites were quantified through HPLC. The data in the plot are representative of three biological replicates, and the error bars indicate mean ± SEM and significance were determined relative to DMSO control (one-way ANOVA with Dunnett’s test ∗*p* < 0.05, ∗∗*p* < 0.01 and ∗∗∗*p* < 0.001 in Graphpad prism 9.3.1). *B*, *C. difficile* R20291 (*A*_600_ ≈ 0.3) was exposed to ENX (*black bars*) or Bz-423 (*red bars*). TcdA quantification was performed on culture supernatants after 24 of exposure. Data from three biological replicates are shown as mean ± SEM (one-way ANOVA with Tukey’s test ∗*p* < 0.05, ∗∗*p* < 0.01 and ∗∗∗*p* < 0.001 done using Graphpad prism version 9.3.1). *C*, gene expression was analyzed by quantifying mRNA in R20291 (n = 3 biological replicates) exposed to 16 μM (1× EC_50_) ENX. Exponentially growing cells (*A*_600_ ≈ 0.3) were exposed to ENX or DMSO for 30 min before RNA was extracted for RNA-seq. *D*, mRNA levels for the genes involved in phosphate metabolism were analyzed by reverse transcriptase quantitative PCR, and the fold change was calculated as the difference in mRNA levels of control *versus* ENX-treated cells; mean ± SEM are shown. Statistical significance was assessed by comparing ΔCt values of DMSO and ENX treatment by two-tailed paired *t*-tests; ∗∗*p* < 0.01 in Graphpad prism 9.3.1. DMSO, dimethyl sulfoxide.

After attempts to purify AtpA for biophysical analysis failed, we conducted target overexpression of *atpA* in R20291, under the Ptet promoter, to out-titrate enoxolone inhibition. However, this did not affect the EC_50_ of enoxolone (*data not shown*) and might reflect that overexpression of a single component of the F1F0-ATP synthase complex may not affect overall protein function. However, HPLC analysis of nucleotide metabolites revealed that enoxolone (16 μM, *i.e.*, EC_50_) reduced cellular ATP by 25 ± 5.3% (Fig. 6*A*). Unlike observations from metabolomics, the HPLC did not detect a significant decrease in ADP (Figs. 4*C* and 6*A*). A prior study indicated that more drastic reductions in cellular ATP content of over 90%, caused by membrane-active agents was bactericidal to *C. difficile* (25). Considering that the EC_50_ of enoxolone did not decrease the viability of R20291 (Fig. S1*E*), the effect of enoxolone on ATP content can be considered moderate.

To further investigate the potential for *C. difficile* ATP synthase as an antivirulence drug target, we tested Bz-423, a benzodiazepine that stops ATP synthesis by binding to the delta-subunit of F1F0-ATPase (30, 31). Bz-423 was chosen primarily because of the potential for future chemical modifications of its benzodiazepine structure (32). At growth permissive concentrations of 8 and 16 μM of Bz-423, cellular ATP was reduced by 23.7 ± 13.1% and 32.6 ± 9.6%, respectively, and TcdA biosynthesis by 84.0 ± 5.0% and 90.6 ± 6.0%, respectively (Fig. 6*B*).

### Transcriptome response to enoxolone

Because enoxolone has an apparent multitargeted action, we adopted RNAseq to identify broader impacts on cell physiology. Seventy genes were differentially expressed, according to criteria false discovery rate (FDR) < 0.01 and log_2_(fold change) > 1, upon exposure to enoxolone (16 μM), of which 32 were downregulated and 38 were upregulated (Fig. 6*C* and Table S3). Reverse transcriptase quantitative PCR (RT-qPCR) validation of 20 differentially expressed genes identified by RNA-seq indicated a Pearson’s correlation of 0.8499 (*p* value < 0.0001) (Fig. S6). The most significantly upregulated genes involved phosphate (*i.e.*, Pi) metabolism genes, *CDR20291_1626* (a putative sodium/phosphate symporter; 4.9-fold) and genes annotated to be part of the Pho regulon (*CDR20291_3120* [encoding a putative phosphate ABC transporter, PstA; 2.4-fold], *CDR20291_3121* [encoding a putative phosphate ABC transporter, PstC; 2.3-fold], *phoT* [CDR20291_3119, also known as *pstB*, encoding a putative *PstB* phosphate import ABC transporter, ATP-binding protein; 2.7-fold] and *phoU* [*CDR20291_3118*, encoding a putative phosphate transport system regulatory protein PhoU; 2.9-fold]) (Table S3). CDR20291_1626 appears to be an ortholog of *Vibrio cholerae* NptA (33), a low affinity sodium dependent phosphate symporter. Genes *CDR20291_3118*, *CDR20291_3119*, *CDR20291_3120*, and *CDR20291_3121* are predicted to be part of an operon. RT-qPCR confirmed that *phoU* transcription was increased by 15.89 ± 3.69–fold in enoxolone (16 μM) (Fig. 6*D*). The gene *CDR20291_3129* that is not part of the operon appears to encode a PstS ortholog and was upregulated by 1.6 fold in the RNAseq (Table S3). Thus, it appears that *C. difficile* encodes a PstSCAB transporter that is known to be a part of the Pho regulon (a gene network that mediates the cellular acquisition, use, and conservation of phosphate) (34).

### Enoxolone depletes cellular phosphates

**Figure 7:**
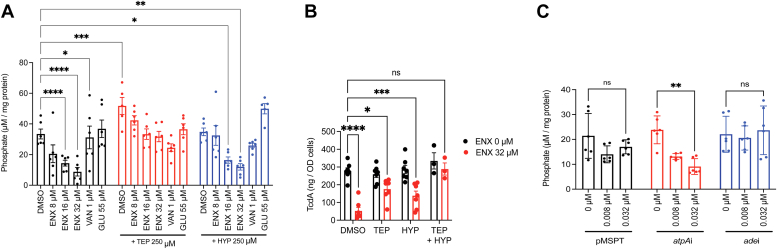
**Analysis of effects of enoxolone and ATP synthase on cellular phosphate (Pi) metabolism and toxin production.***A*, *Clostridioides difficile* at *A*_600_ ≈ 0.3 was treated with 8, 16, or 32 μM of enoxolone (ENX), or 1 μM of vancomycin (VAN), or 55 μM of glucose (GLU) and harvested after 3 h. Intracellular Pi was analyzed from whole cell lysates (n = 3 biological replicates; with exception of GLU/HYP and DMSO/TEP, all other samples had two technical replicates within the three biological replicates). Significance was assessed relative to DMSO control by two-way ANOVA with Dunnett’s test ∗*p* < 0.05, ∗∗*p* < 0.01, ∗∗∗*p* < 0.001, and ∗∗∗∗*p* < 0.0001; in Graphpad prism 9.3.1. *B*, TcdA quantification from 24 h old cultures of *C. difficile* R20291 cells exposed to 250 μM metabolite in presence of DMSO (*black bars*) or 32 μM ENX (*red bars*). The data in the plot are representative of minimum three biological replicates, and the error bars indicate mean ± SEM and the data significance is representative of two-way ANOVA with Tukey’s test ∗*p* < 0.05, ∗∗*p* < 0.01, and ∗∗∗*p* < 0.001 in Graphpad prism 9.3.1. *C*, relationship between AtpA activity and cellular phosphates, determined from phosphate levels in *C. difficile* R20291 cells carrying empty vector or the vector encoding an antisense RNA (asRNA) to *atpA* or *ade*. Cells were grown to *A*_600_ ≈ 0.3, and asRNA induced with indicated concentrations of ATc. Cells were harvested after 3 h of exposure to ATc and their phosphate contents were measured from whole cell lysates (n = 3 biological and two technical replicates; effects of gene silencing on toxin production are in Figs. 3 and S4). Significance was assessed for phosphate levels relative to uninduced cells (ATc 0 μg/ml) by two-way ANOVA with Tukey’s test ∗∗*p* < 0.01 in Graphpad prism 9.3.1. ade, adenine deaminase; ATc, anhydrotetracycline; atpA, ATP synthase subunit alpha; DMSO, dimethyl sulfoxide; TEP, triethyl phosphate.

Activation of genes involved in phosphate uptake suggested that enoxolone perturbed intracellular phosphate (Pi) levels. To test this, we quantified intracellular Pi in cells exposed to enoxolone for 3 h. Results showed that enoxolone depleted intracellular Pi in a dose-dependent manner (Fig. 7*A*), with 16 and 32 μM of compound causing decreases of 54.6 ± 17.6% and 75.6 ± 29.5%, respectively. For comparison, cellular Pi pools were not affected by vancomycin and glucose (Fig. 7*A*).

To determine whether cellular Pi impacted toxin production, we supplemented the growth medium with either a phosphate source (triethyl phosphate, TEP) or hypoxanthine. In the presence of enoxolone (32 μM, *i.e.*, 2× EC_50_), TEP restored intracellular Pi (Fig. 7*A*) and increased toxin production by ∼60% (Fig. 7*B*). Conversely, hypoxanthine did not restore cellular Pi, suggesting that the effects of enoxolone on purine metabolism were unlikely to impact cellular Pi pools.

We next analyzed the relationship between cellular Pi and ATP, in the presence of enoxolone (2× EC_50_, 32 μM). Firstly, supplementation with TEP restored ATP levels in the presence of enoxolone (Fig. 6*A*). Conversely, hypoxanthine, which did not affect Pi levels, also did not affect cellular ATP (Fig. 6*A*). Since these observations suggest enoxolone affected two independent pathways we tested whether supplementation of both TEP and hypoxanthine was required to fully restore toxin A production. As shown in Figure 7*B* both substrates were required to bypass the effect of enoxolone on toxin production.

### Silencing *atpA* depleted cellular phosphates

Since enoxolone depleted cellular Pi, we next determined if it could be mediated through *atpA* or *ade*. We therefore quantified cellular Pi in cells expressing antisenses to *atpA* or *ade*. Silencing *atpA* mRNA caused depletion of cellular Pi in an ATc concentration-dependent manner (Fig. 7*C*) *i.e.*, ATc at 0.008 and 0.032 μg/ml reduced cellular phosphate by 36.33 ± 17.2% and 56.14 ± 15.75%, respectively. Conversely, silencing *ade* had no effect on cellular Pi (Fig. 7*C*). These results suggest that the activity of ATP synthase affected cellular amounts of Pi. This observation is consistent with previous findings that in *Salmonella enterica* serovar Typhimurium the deletion of the ATPase beta subunit (*atpB*) caused a reduction in cellular Pi levels (35).

## Discussion

From screening of structurally diverse phytochemicals, we identified that the pentacyclic triterpenoid enoxolone (18*β*-glycyrrhetinic acid) inhibits toxin biosynthesis in *C. difficile*, by disrupting multiple metabolic pathways. Its discovery and the results presented here provide a framework for unbiasedly identifying inhibitors of *C. difficile* virulence and their use as chemical genetic probes to uncover pathways affecting the biosynthesis of TcdA and TcdB. Such approaches may help to overcome bottlenecks in clostridial genetics. For instance, unlike current clostridial genetic tools, chemical probes may be applied to interrogate the metabolic and genetic responses across heterogenetic lineages of *C*. *difficile* (36).

Against R20291, enoxolone inhibited TcdA and TcdB biosynthesis at an EC_50_ of 16 μM and partly impacted growth at higher concentrations (≥32 μM). Through multiomics, genetics, and biochemical approaches we discovered and validated that enoxolone impedes purine, phosphate, and ATP metabolisms, suggesting it has a multitarget action. This is consistent with reports that triterpenoids interact with multiple cellular targets (37, 38). The targets identified and validated were Ade and AtpA. In the purine salvage pathway, Ade converts adenine to hypoxanthine, which is eventually metabolized to guanine nucleotides either *via* a xanthine intermediate or inosine (39). Supporting metabolomic studies showed enoxolone reduced cellular hypoxanthine content, while SPR and ITC corroborated that enoxolone binds to Ade. Accordingly, our findings with Ade suggest that additional research is warranted to determine whether other proteins within *C. difficile* purine salvage pathway might be potential antivirulence drug targets. Similar therapeutic concepts have been explored in other pathogens. For instance, the *de novo* and salvage pathways converge at steps catalyzed by GuaB (IMP dehydrogenase), GuaA (GMP synthase), or Gmk (guanylate kinase), and disruption of *guaB* and *guaA* attenuates virulence in *Yersinia pestis* (40), *Francisella tularensis* (41), and *Staphylococcus aureus* (42).

Retardation of *C. difficile* growth by enoxolone can be explained by two likely scenarios. Firstly, the cellular build-up of adenine might be toxic to *C. difficile* since adenine supplementation attenuated growth (Fig. 5*A*). It is also plausible that Ade inhibition affected downstream synthesis of guanosine phosphates, which in turn might have reduced growth *via* multiple mechanisms. For example, one mechanism might be cell division, since GTP exhaustion is known to prevent polymerization of FtsZ, a cell division protein (43) that is sensitive to changes in purine metabolism (44). Although ATP depletion is also known to affect growth, TEP supplementation that restored ATP levels did not enhance growth unlike xanthine and guanine supplementations (Fig. S7). Thus, inhibition of purine metabolism affected both growth and toxin biosynthesis, whereas decreases in cellular ATP and Pi mainly appeared to affect toxin biosynthesis.

Considering data from gene silencing of *atpA*, enoxolone’s modest depletion of ATP content (∼25%), metabolite analysis, and the reduction of toxin production by Bz-423, there is an apparent correlation between ATP production *via* ATP synthase and toxin biosynthesis in *C. difficile*. We also observed that enoxolone and silencing of *atpA* reduced cellular Pi. A similar observation was also seen in *S. enterica* serovar Typhimurium where loss of *atpB* decreased cellular Pi pools, thus linking ATP synthase activity with the regulation of Pi uptake (35). Interestingly, a Tn-Seq screen (45) suggested that *atpA* is essential for *C. difficile* growth, which disagrees with our findings. Although Tn-Seq is an effective method for examining gene essentiality in bacterial genomes, it also generates false-positive and false-negative results that require genetic validation, through gene knockouts, gene silencing, or metabolic analysis (46, 47). Moreover, in different bacteria the loss of ATP synthase is nonlethal; for example, in *Clostridium acetobutylicum* genetic disruption of ATP synthase gamma chain (*atpG*) did not impact growth (48); and in *E. coli* (49), *S. enterica* (Typhimurium) (35), and *S. aureus* (50) deletion of ATP synthase appears to be conditionally nonessential.

**Figure 8:**
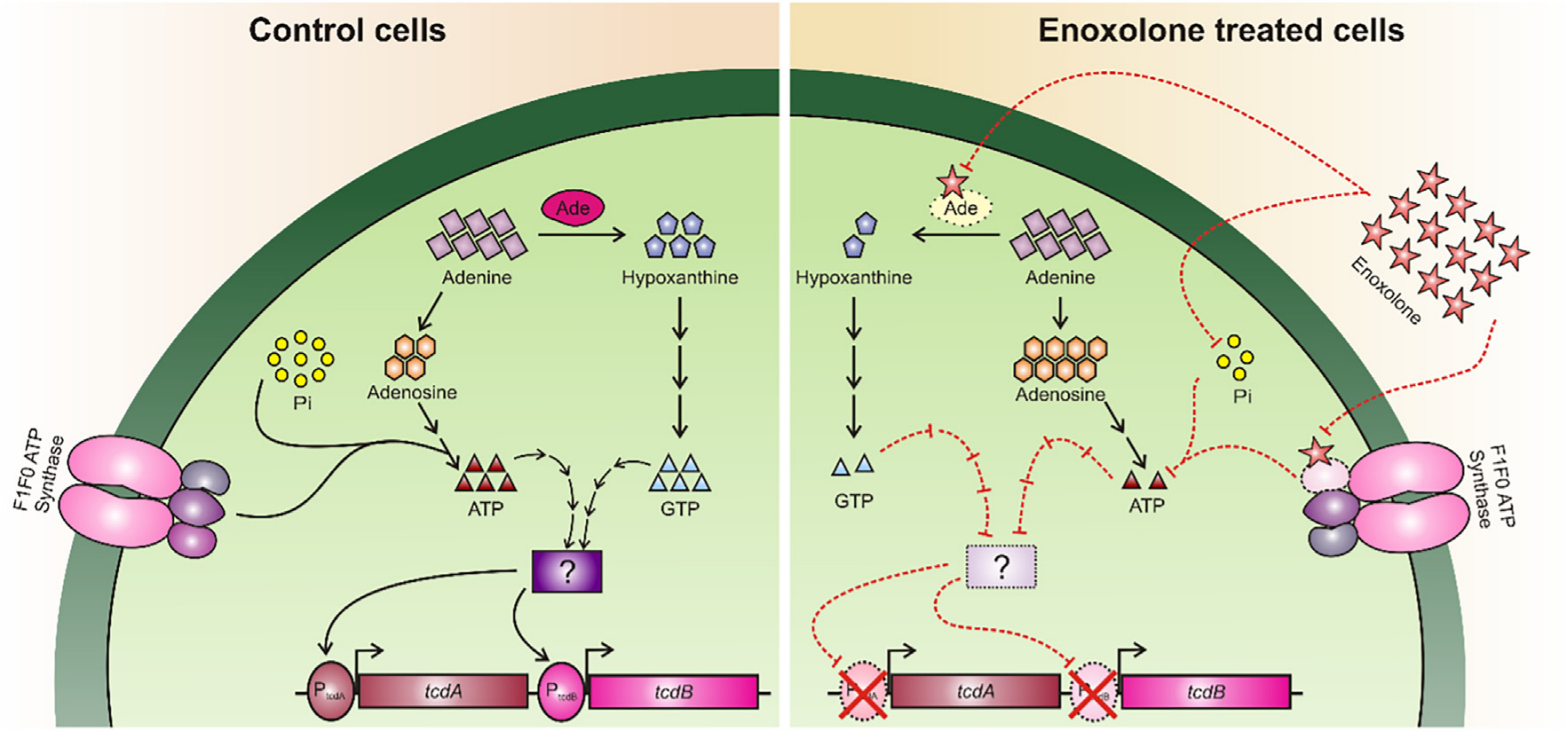
**Proposed model for enoxolone mode of action against *Clostridioides difficile*.** (*Left*) scheme depicting the molecular events during the regular growth of *C. difficile*. (*Right*) represents adaptation of *C. difficile* to enoxolone, which hampers multiple processes in cells including ATP, phosphate, and purine metabolisms. The protein designated with a question mark, represents an unknown effector that might be a downstream factor causing repression of toxin genes. While effectors of toxin gene expression are known (*e.g.*, TcdR, CcpA, and CodY), it is not unknown whether enoxolone suppression of toxin production works through these or other regulators. CcpA, catabolite control protein A.

Our study has limitations, such as AtpA could not be purified for molecular interaction research, as was done for Ade, as well as cellular pools of guanosine phosphates was not quantifiable (*data not shown*). In addition, the interpretation of *in vivo* results of enoxolone efficacy in mice with CDI was complicated by the compound causing adverse side effects (Fig. S8), namely weight loss and its well-documented antiinflammatory properties (reduction of tumor necrosis factor-alpha and interleukin-6 synthesis) (51, 52). We must emphasize that enoxolone is not a drug candidate; rather, it might be a starting point for chemical modification or for safer pentacyclic triterpenoid analogs to be used to manage CDI. Despite these limitations, our findings extend emerging knowledge that disruption of energy metabolism affects *C. difficile* virulence (53). In conclusion, as depicted in the mechanistic model of enoxolone action (Fig. 8), Ade and F0F1-type ATP synthase are potentially druggable antivirulence targets. This warrants further target validation studies, particularly in CDI animal models to assess the pathogenesis of loss-of-function mutants of Ade and FoF1-type ATP synthase, and to determine the extent to which intestinal purines or phosphates are compensatory (54, 55, 56).

## Experimental procedures

### Bacteria and growth conditions

*C. difficile* strains were grown in brain heart infusion (BHI) agar or broth and when needed supplemented with 0.1% (w/v) taurocholate or thiamphenicol (15 μg/ml). Anaerobic cultures were grown at 37 °C in anaerobic chamber (Don Whitley A35 anaerobic chamber). *E. coli* was grown aerobically at 37 °C in Luria Bertani agar or broth with kanamycin (35 μg/ml), when appropriate.

### Growth kinetics

Growth kinetics in BHI broth were determined as described previously (53), using exponentially growing cells at *A*_600_ ≈ 0.2. Automated growth was recorded every 60 min for 20 h with shaking before each read in an Infinite M Plex microplate reader (Tecan) in an anaerobic chamber (Coy Laboratory Products).

### Toxin quantification

Single colonies of *C. difficile* were inoculated into 5 ml of BHI broth. The resulting overnight culture was subcultured into fresh BHI medium at 1% v/v and grown to *A*_600_ ≈ 0.2 to 0.3, before compounds or supplements were added to desired final concentrations. After 24 h of incubation cell densities (*A*_600_ nm) were recorded with VersaMax Microplate reader (Molecular Devices). After centrifugation, supernatants were collected and stored at −80 °C for toxin quantification by cell rounding or ELISA, as described below.

#### Cell rounding assay

Vero cells (50 μl) from the American Type Culture Collection were aliquoted in 384 well plates at a density of 10^4^ cells/ml and incubated overnight at 37 °C in a CO_2_ incubator. Culture supernatants (100 nl) were added to the Vero cells with an Echo acoustics liquid handler (Labcyte) and the plates incubated at 37 °C in a CO_2_ incubator for 3 h and then fixed with 4% (v/v) paraformaldehyde. After washing twice using a HydroSpeed washer (Tecan), cells were stained with 4′,6-diamidino-2-phenylindole (3.5 μM) for 15 min, washed and stained with 6 nM of Alexa Fluor 488 phalloidin stain (Invitrogen) for 2 h. Cells were then washed twice, before PBS (50 μl) was added. Images were recorded with an IN Cell Analyzer 6000 (GE HealthCare) for 4′,6-diamidino-2-phenylindole (excitation 405 nm and emission 455 nm) and Alexa Fluor 488 phalloidin (excitation 495 nm and emission 515 nm). Images were acquired from four fields/well at 10× magnification and exposure time of 1500 ms.

Image analysis was performed by calculating morphometric features using advanced imaging library in Pipeline pilot version 9.2 (Biovia). In brief, the background was corrected, followed by segmenting the nuclei and cell boundaries using thresholding and a watershed to separate neighboring objects. A panel of morphometric features was then extracted for both the nuclear and cellular mask. We trained a Random Forest classifier on a 50:50 randomly subsampling morphometric data from 16 toxins treated and 16 nontreated control wells, which were used as pseudo-labels for the cellular condition. Statistical evaluation of the model showed an area under the receiver operator characteristic curve for the out-of-bag training set to be 0.95. Gini analysis showed that equivalent diameter, area, and convex area are the most important features used in classification. Confusion matrix analysis and other descriptive statistics of the with-held test set show a balanced accuracy of 0.93, indicating the model was robust. Given these observations, this model was applied to all morphometric analysis and reported as the proportion of rounded cells *versus* the total number of cells.

#### Toxin quantification by ELISA

TcdA and TcdB were quantified with *C. difficile* toxin A or B ELISA kit (tgcBIOMICS), according to the manufacturer’s instructions.

### Sporulation assay

This assay was performed as described previously (24). Briefly, cultures were grown anaerobically until *A*_600_ ≈ 0.3, and exposed to compound for 5 days before enumerating total viable counts and spores.

### Synthesis of enoxolone click molecule, compound 3511

Compound 3511 was synthesized by mixing 1 mmol of enoxolone with 1.1 mmol each of propargylamine and 4-(4,6-dimethoxy-1,3,5-triazin-2-yl)-4-methylmorpholinium chloride in 10 ml methanol by stirring at room temperature for 5 h. After evaporation of methanol, the residue was dissolved in 10 ml dichloromethane and sequentially extracted with 10 ml each of saturated aqueous NaHCO_3_, water, 5% citric acid, water, brine, dried over Na_2_SO_4_, and concentrated. The residue was purified by column chromatography using hexanes/ethyl acetate (3:1–1:1) to provide 241 mg (42%) of product as white solid. ^1^H NMR (400 MHz, CDCl_3_) δ 5.75 (t, *J* = 5.1 Hz, 1H), 5.62 (s, 1H), 4.10–3.90 (m, 2H), 3.16 (dd, *J* = 10.6, 5.7 Hz, 1H), 2.72 (dt, *J* = 13.5, 3.6 Hz, 1H), 2.27 (s, 1H), 2.18 (t, *J* = 2.5 Hz, 1H), 2.15–2.05 (m, 1H), 2.03–1.91 (m, 1H), 1.90–1.46 (m, 10H), 1.45–1.29 (m, 8H), 1.16–1.09 (m, 1H), 1.09–1.04 (m, 8H), 1.02–0.82 (m, 5H), 0.74 (d, *J* = 5.5 Hz, 6H), 0.63 (dd, *J* = 11.8, 1.9 Hz, 1H). ^13^C NMR (101 MHz, CDCl_3_) *δ* 200.15, 175.49, 169.02, 128.55, 79.74, 78.79, 71.70, 61.84, 54.97, 48.04, 45.37, 43.54, 43.21, 41.83, 39.18, 39.14, 37.36, 37.11, 32.78, 31.91, 31.46, 29.33, 29.30, 28.40, 28.11, 27.30, 26.48, 26.41, 23.36, 18.70, 17.49, 16.37, 15.57. Mass spectrometry-electrospray ionisation (ESI), m/z = 508.38 [M + H]^+^.

### Click chemistry, affinity enrichment, and protein identification

Overnight cultures of R20291 were diluted 1:100 into fresh BHI broth and grown to *A*_600_ ≈ 0.5. Cells were harvested by centrifugation at 4000*g* for 15 min. The cell pellet was resuspended in buffer A (50 mM potassium phosphate pH 7.4, 150 mM NaCl) and supplemented with 10 mM MgCl_2_, 100 μg/ml of DNaseI, and One cOmplete Protease Inhibitor (Roche). After cell lysis with a cell disruptor (Constant Systems Ltd), the lysates were centrifuged (10,000*g* for 15 min at 4 °C). Membrane vesicles were isolated by centrifugation (100,200*g* for 25 min at 4 °C) and the supernatant (cytosolic fraction) was collected. The membrane pellet was resuspended in buffer A with 10% (v/v) glycerol. Both the cytosolic and the membrane protein samples were aliquoted and stored at −80 °C until use. Protein concentrations were quantified using bicinchoninic acid protein assay (Thermo Fisher Scientific), with bovine serum albumin as the calibration standard.

Click reaction between compound 3511 and TAMRA-biotin-azide (Click Chemistry tools) was performed in presence of 100 μM CuSO4, 500 μM tris-hydroxypropyltriazolylmethylamine and 5 mM Na-ascorbate. After incubation for 2 h in the dark at room temperature, the reaction was supplemented with cytosolic or membrane fractions (1 mg/ml) and incubated further for 4 h at 4 °C. The clicked protein samples were then analyzed on 4 to 20% tris-glycine gradient gels (NuSep) and the TAMRA-biotin-azide conjugated protein samples were visualized by in-gel fluorescence using FluorChem M system (ProteinSimple), with excitation 550 nm and emission 570 nm.

For the affinity pull-downs, 100 μl of streptavidin magnetic beads (Roche) were washed thrice with 10 volumes of buffer A. Protein samples were added to the beads and incubated overnight at 4 °C on a rotating shaker (Barnstead Thermolyne). Next the beads were washed five times with ten volumes of buffer A and bound proteins eluted with 0.1 M glycine-HCl, pH 2.5. Entire eluted samples were subjected to liquid chromatography with tandem mass spectrometry at the Proteomics Service Center, University of Texas Health Science Center.

### Plasmid construction

Antisense fragments (100 bp), cloned into the anhydrotetracycline inducible vector pMSPT, were designed to target the ribosome binding site and start codon region as described (24). For overexpressing Ade protein, the codon optimized *ade* gene was cloned within the *Bam*HI/*Xho*I sites of pWL613a plasmid which is derived from pET28b by PPCMI core at Institute for Biosciences and Technology, Texas A&M.

### Protein expression and purification

To purify Ade, codon optimized *ade* was cloned into pWL613a, a derivative of pET28b plasmid, by standard cloning techniques and transformed into *E. coli* expression strain Bl21 (DE3). The strain was grown in LB medium with 50 μg/ml of kanamycin at 37 °C until the *A*_600_ ≈ 0.8. Cultures were cooled to 20 °C, and protein expression induced with 500 μM IPTG for 16 h. Cells were harvested, lysed, and soluble cytosolic fractions were collected following cell lysis with a cell disruptor. Ade was purified from Ni-NTA agarose beads (Marvelgent Biosciences Inc) following preequilibration with buffer A (20 mM Tris pH 8.0 with 150 mM NaCl) containing 10 mM imidazole, pH 8.0 for 1 h, at 4 °C on a rotating shaker (Barnstead Thermolyne). Proteins were eluted from Ni-NTA columns with buffer A containing 300 mM imidazole, pH 8.0 and further purified by size-exclusion chromatography on a Superdex 200 column (GE HealthCare) equilibrated with buffer A.

### Surface plasmon resonance

The binding of enoxolone to adenine deaminase protein was analyzed using Biacore T200 SPR machine (GE HealthCare), using a carboxymethylated CM5 chip (Cytiva). The CM5 chip was activated with 180 μl mixture of 10 mM NHS (*N*-hydroxysuccinimide) and ∼39 mM EDC (1-Ethyl-3-(3-dimethylaminopropyl) carbodiimide hydrochloride) at a flowrate of 7 μl/min. Monoclonal antihistidine antibody was immobilized onto chip by passing 50 μg/ml anti-His antibody in 10 mM sodium acetate, pH 4.5 at a flowrate of 7 μl/min for 7 min (7000–15,000 RU). The recombinant Ade protein (100 μg/ml) was injected for trapping onto the chip through the immobilized anti-His antibody, with a contact time of 90 s at a flow rate of 10 μl/min. The binding experiments were performed by injecting test compounds in 20 mM Tris pH 7.4 with 150 mM NaCl, 1% (v/v) DMSO and 0.1% (w/v) bovine serum albumin at a constant temperature of 25 °C. The anti-His antibody was regenerated by rinsing with 10 mM glycine pH 1.5, with a contact time of 30 s at a flow rate of 30 μl/min. Solvent correction was done with a seven-point solvent correction with DMSO concentrations ranging from 0.5% to 2.0% (all other buffer conditions were kept constant). Affinity values were determined using Biacore T200 Evaluation Software from Cytia.

### Analysis of cellular metabolite concentrations

Overnight cultures of *C. difficile* R20291 were diluted 1:100 into fresh BHI broth. Cultures were grown to *A*_600_ ≈ 0.3, and compounds added to desired concentrations. After 3 h the cultures were supplemented with equal volumes of chilled methanol and incubated at −20 °C for 5 min. Samples were harvested by centrifugation (4000*g* for 10 min), washed with chilled buffer of 20 mM Tris, 100 mM NaCl, pH 8.0. Cell pellets were stored at −80 °C until further processing.

### Metabolome analysis by mass spectrometry

Cells were processed as previously described (57) and extracted samples analyzed by HPLC coupled to Agilent 6495 Triple Quadrupole mass spectrometry, using ESI positive ionization and single reaction monitoring mode. Nucleotides were identified in ESI positive mode using Zorbax eclipse XDB C-18, 1.8-micron, 4.6 × 100 mm column. Mobile phases A and B were 0.1% formic acid in water and acetonitrile, respectively. The gradients used are as follows: 0 min 2% of B; 6 min 2% of B, 6.5 min 30% of B, 7 min 90% of B, 12 min 95% of B, and 13 min 2% of B followed by reequilibration at the end of the gradient 20 min to the initial starting condition 2% of B. Flow rate: 0.2 ml/min. The data were processed using Mass Hunter Quantitative software from Agilent. The data were normalized with internal standard and log_2_-transformed per-sample, per-method basis. For every metabolite in the normalized dataset, two sample *t* tests were conducted to compare expression levels between different groups. Differential metabolites were identified by adjusting the *p*-values for multiple testing at an FDR threshold of <0.25 and generated a heat map.

### HPLC quantification of adenosine nucleotides

Cells were resuspended in 400 μl of 0.1 M perchloric acid, followed by disruption by probe sonication for 1 min (pulses for 5 s on 50% duty cycle using Q125 Sonicator (Qsonica)). Cell lysates were centrifuged (10,000*g* for 10 min) and supernatants treated with 2.5% (v/v) of 3.5 M potassium chromate. Samples were filtered through 0.45 μM polyvinylidene difluoride membranes and analyzed by HPLC for purine metabolites at HPLC Bioanalytical Core at Emory University.

### RNA extraction and sequencing

R20291 cultures (*A*_600_ ≈ 0.3) were supplemented with either DMSO or enoxolone (16 μM). After 30 min, one volume of RNAprotect bacterial reagent (Qiagen) was added followed by centrifugation (4000*g* for 10 min). Cells were resuspended in 700 μl of Qiazol lysis reagent (Qiagen) and lysed in a FastPrep cell disruptor (Qiagen) for 5 min at force 50. Total RNA was extracted using the RNeasy Mini Kit (Qiagen), according to the manufacturer’s instructions. Integrity of RNA was estimated on an Agilent 2100 Bioanalyzer with all samples possessing an RNA integrity number >7. Ribosomal RNA was depleted with NEBNext rRNA Depletion Kit (New England Biolabs). Libraries were prepared from ribosomal-depleted RNA with the TruSeq Stranded mRNA Library Prep Kit according to the manufacturer’s instructions (Illumina, PN 20020595). Libraries were analyzed for insert size distribution using the 2100 BioAnalyzer High Sensitivity kit (Agilent Technologies). Libraries were quantified using the Quant-iT PicoGreen ds DNA assay (Thermo Fisher Scientific) and by low pass sequencing with a MiSeq nano kit (Illumina). Paired end 100 cycle sequencing was performed on a NovaSeq 6000 (Illumina) at the Hartwell Center at St Jude Children’s Research Hospital.

### Transcriptome analysis

The resulting reads were saved in FASTQ format and data were processed through the High-Performance Computing Facility at St Jude. Adapter content and quality trimming was performed using Cutadapt (58). The quality of the raw and trimmed reads was assessed using FastQC and MultiQC (59). The resulting processed reads were aligned to the reference *Clostridium difficile* R20291 genome (Genome accession: FN545816) using STAR (60) and aligned reads were counted using featureCounts (61). Differential gene expression analysis was analyzed using DESeq2 (62) in the R statistical computing environment with significant differentially expressed genes determined using an FDR cutoff of <0.01 and an absolute value of log_2_(FC) >1. The raw and processed RNA-seq data associated with this study have been deposited to NCBI GEO under accession number GSE199109.

### Gene expression analysis by RT-qPCR

The quantification of transcripts for various genes were analyzed by RT-qPCR as described previously (63) with slight modifications. The RNA samples were prepared as indicated above and the complementary DNAs were synthesized using qScript cDNA supermix (Quanta Biosciences) and RT-qPCR done with qScript SYBR Green RT-qPCR master mix (Quanta Biosciences) in Applied Biosystems ViiA7 RT-PCR system. Results were calculated using the 2^−ΔΔCt^ method and transcript levels normalized to 16S rRNA.

### Phosphate quantification

Overnight cultures of *C. difficile* strains were diluted by 20-fold into a fresh medium and grown until *A*_600_ ≈ 0.3. compounds or metabolites were added to the cultures at indicated concentrations and the cultures were incubated for 3 h. Cells were harvested by centrifugation (4000*g* for 10 min) followed by washing cells five times with MilliQ water. Cell pellets were resuspended in 1 ml of ice cold MilliQ water, and the cells were lysed by FastPrep (Qiagen) for 5 min, and the samples were centrifuged at 21,100*g* for 5 min. Pi content was measured using the Malachite Green Phosphate Assay Kit (Sigma-Aldrich) according to the manufacturer’s instructions.

### CDI colitis mouse model

Animal studies involving treatment of C57BL/6 mice infected with *C. difficile* are fully described in Figure 8 and were conducted under an animal use protocol approved by The Institutional Animal Care and Use Committee of Texas A&M University. Experimental details are described in the legend of Figure 8.

## Data availability

All data in this manuscript are described in the figures or accompanying Supporting information. The raw and processed RNA-seq data associated with this study have been deposited to NCBI GEO under accession number GSE199109. Data relating to effect of ATP synthase overexpression on activity of enoxolone or ineffective quantification of GTP are not shown but can be attained by emailing the corresponding author (JGH, jhurdle@tamu.edu). We have no exceptions or limitations on sharing of data and materials from this study.

## Supporting information

This article contains supporting information (51, 52, 64, 65, 66, 67, 68, 69, 70, 71, 72).

## Conflict of interest

The authors declare that they have no conflicts of interest with the contents of this article.

## References

[1] Guh A.Y., Mu Y., Winston L.G., Johnston H., Olson D., Farley M.M. (2020). Trends in U.S. burden of *Clostridioides difficile* infection and outcomes. N. Engl. J. Med..

[2] Leffler D.A., Lamont J.T. (2015). *Clostridium difficile* infection. N. Engl. J. Med..

[3] Kelly C.P. (2012). Can we identify patients at high risk of recurrent *Clostridium difficile* infection?. Clin. Microbiol. Infect..

[4] Lewis B.B., Buffie C.G., Carter R.A., Leiner I., Toussaint N.C., Miller L.C. (2015). Loss of microbiota-mediated colonization resistance to *Clostridium difficile* infection with oral vancomycin compared with metronidazole. J. Infect. Dis..

[5] Zhang Y., Limaye P.B., Renaud H.J., Klaassen C.D. (2014). Effect of various antibiotics on modulation of intestinal microbiota and bile acid profile in mice. Toxicol. Appl. Pharmacol..

[6] Martin-Verstraete I., Peltier J., Dupuy B. (2016). The regulatory networks that control *Clostridium difficile* toxin synthesis. Toxins (Basel).

[7] Gerding D.N., Johnson S. (2010). Management of *Clostridium difficile* infection: thinking inside and outside the box. Clin. Infect. Dis..

[8] Gerding D.N., Kelly C.P., Rahav G., Lee C., Dubberke E.R., Kumar P.N. (2018). Bezlotoxumab for prevention of recurrent *C. difficile* infection in patients at increased risk for recurrence. Clin. Infect. Dis..

[9] Paparella A.S., Aboulache B.L., Harijan R.K., Potts K.S., Tyler P.C., Schramm V.L. (2021). Inhibition of *Clostridium difficile* TcdA and TcdB toxins with transition state analogues. Nat. Commun..

[10] Antunes A., Camiade E., Monot M., Courtois E., Barbut F., Sernova N.V. (2012). Global transcriptional control by glucose and carbon regulator CcpA in *Clostridium difficile*. Nucleic Acids Res..

[11] Antunes A., Martin-Verstraete I., Dupuy B. (2011). CcpA-mediated repression of *Clostridium difficile* toxin gene expression. Mol. Microbiol..

[12] Dupuy B., Sonenshein A.L. (1998). Regulated transcription of *Clostridium difficile* toxin genes. Mol. Microbiol..

[13] Dineen S.S., Villapakkam A.C., Nordman J.T., Sonenshein A.L. (2007). Repression of *Clostridium difficile* toxin gene expression by CodY. Mol. Microbiol..

[14] Karlsson S., Burman L.G., Akerlund T. (1999). Suppression of toxin production in *Clostridium difficile* VPI 10463 by amino acids. Microbiology.

[15] Thanissery R., Zeng D., Doyle R.G., Theriot C.M. (2018). A small molecule-screening pipeline to evaluate the therapeutic potential of 2-aminoimidazole molecules against *Clostridium difficile*. Front. Microbiol..

[16] Lv Z., Peng G., Liu W., Xu H., Su J. (2015). Berberine blocks the relapse of *Clostridium difficile* infection in C57BL/6 mice after standard vancomycin treatment. Antimicrob. Agents Chemother..

[17] Wu X., Alam M.Z., Feng L., Tsutsumi L.S., Sun D., Hurdle J.G. (2014). Prospects for flavonoid and related phytochemicals as nature-inspired treatments for *Clostridium difficile* infection. J. Appl. Microbiol..

[18] Lewis K., Ausubel F.M. (2006). Prospects for plant-derived antibacterials. Nat. Biotechnol..

[19] Stabler R.A., Gerding D.N., Songer J.G., Drudy D., Brazier J.S., Trinh H.T. (2006). Comparative phylogenomics of *Clostridium difficile* reveals clade specificity and microevolution of hypervirulent strains. J. Bacteriol..

[20] Torres J., Camorlinga-Ponce M., Munoz O. (1992). Sensitivity in culture of epithelial cells from rhesus monkey kidney and human colon carcinoma to toxins A and B from *Clostridium difficile*. Toxicon.

[21] Xie J., Zorman J., Indrawati L., Horton M., Soring K., Antonello J.M. (2013). Development and optimization of a novel assay to measure neutralizing antibodies against *Clostridium difficile* toxins. Clin. Vaccine Immunol..

[22] Tang H., Porras G., Brown M.M., Chassagne F., Lyles J.T., Bacsa J. (2020). Triterpenoid acids isolated from *Schinus terebinthifolia* fruits reduce *Staphylococcus aureus* virulence and abate dermonecrosis. Sci. Rep..

[23] Wahab S., Annadurai S., Abullais S.S., Das G., Ahmad W., Ahmad M.F. (2021). *Glycyrrhiza glabra* (licorice): a comprehensive review on its phytochemistry, biological activities, clinical evidence and toxicology. Plants (Basel).

[24] Marreddy R.K.R., Wu X., Sapkota M., Prior A.M., Jones J.A., Sun D. (2019). The fatty acid synthesis protein enoyl-acp reductase II (FabK) is a target for narrow-spectrum antibacterials for *Clostridium difficile* infection. ACS Infect. Dis..

[25] Wu X., Cherian P.T., Lee R.E., Hurdle J.G. (2013). The membrane as a target for controlling hypervirulent *Clostridium difficile* infections. J. Antimicrob. Chemother..

[26] Majumdar A., Govind R. (2022). Regulation of *Clostridioides difficile* toxin production. Curr. Opin. Microbiol..

[27] Neumann-Schaal M., Jahn D., Schmidt-Hohagen K. (2019). Metabolism the difficile way: the key to the success of the pathogen *Clostridioides difficile*. Front. Microbiol..

[28] Kamat S.S., Bagaria A., Kumaran D., Holmes-Hampton G.P., Fan H., Sali A. (2011). Catalytic mechanism and three-dimensional structure of adenine deaminase. Biochemistry.

[29] Moffatt B.A., Ashihara H. (2002). Purine and pyrimidine nucleotide synthesis and metabolism. Arabidopsis Book.

[30] Johnson K.M., Chen X., Boitano A., Swenson L., Opipari A.W., Glick G.D. (2005). Identification and validation of the mitochondrial F1F0-ATPase as the molecular target of the immunomodulatory benzodiazepine BZ-423. Chem. Biol..

[31] Starke I., Glick G.D., Borsch M. (2018). Visualizing mitochondrial F_0_F_1_-ATP synthase as the target of the immunomodulatory drug BZ-423. Front. Physiol..

[32] Huang Y., Khoury K., Chanas T., Domling A. (2012). Multicomponent synthesis of diverse 1,4-benzodiazepine scaffolds. Org. Lett..

[33] Lebens M., Lundquist P., Soderlund L., Todorovic M., Carlin N.I. (2002). The nptA gene of *Vibrio cholerae* encodes a functional sodium-dependent phosphate cotransporter homologous to the type II cotransporters of eukaryotes. J. Bacteriol..

[34] Santos-Beneit F. (2015). The *Pho* regulon: a huge regulatory network in bacteria. Front. Microbiol..

[35] Bruna R.E., Kendra C.G., Groisman E.A., Pontes M.H. (2021). Limitation of phosphate assimilation maintains cytoplasmic magnesium homeostasis. Proc. Natl. Acad. Sci. U. S. A..

[36] Bustin K.A., Abbas A., Wang X., Abt M.C., Zackular J.P., Matthews M.L. (2023). Characterizing metabolic drivers of *Clostridioides difficile* infection with activity-based hydrazine probes. Front. Pharmacol..

[37] Kuo R.Y., Qian K., Morris-Natschke S.L., Lee K.H. (2009). Plant-derived triterpenoids and analogues as antitumor and anti-HIV agents. Nat. Prod. Rep..

[38] Hansen J., Palmfeldt J., Vang S., Corydon T.J., Gregersen N., Bross P. (2011). Quantitative proteomics reveals cellular targets of celastrol. PLoS One.

[39] Petersen C., Moller L.B., Valentin-Hansen P. (2002). The cryptic adenine deaminase gene of *Escherichia coli*. Silencing by the nucleoid-associated DNA-binding protein, H-NS, and activation by insertion elements. J. Biol. Chem..

[40] Oyston P.C., Mellado-Sanchez G., Pasetti M.F., Nataro J.P., Titball R.W., Atkins H.S. (2010). A *Yersinia pestis guaBA* mutant is attenuated in virulence and provides protection against plague in a mouse model of infection. Microb. Pathog..

[41] Santiago A.E., Cole L.E., Franco A., Vogel S.N., Levine M.M., Barry E.M. (2009). Characterization of rationally attenuated *Francisella tularensis* vaccine strains that harbor deletions in the *guaA* and *guaB* genes. Vaccine.

[42] Kofoed E.M., Yan D., Katakam A.K., Reichelt M., Lin B., Kim J. (2016). *De novo* guanine biosynthesis but not the riboswitch-regulated purine salvage pathway is required for *Staphylococcus aureus* infection *in vivo*. J. Bacteriol..

[43] Mukherjee A., Lutkenhaus J. (1998). Dynamic assembly of *ftsZ* regulated by GTP hydrolysis. EMBO J..

[44] Jones-Carson J., Yahashiri A., Kim J.S., Liu L., Fitzsimmons L.F., Weiss D.S. (2020). Nitric oxide disrupts bacterial cytokinesis by poisoning purine metabolism. Sci. Adv..

[45] Dembek M., Barquist L., Boinett C.J., Cain A.K., Mayho M., Lawley T.D. (2015). High-throughput analysis of gene essentiality and sporulation in *Clostridium difficile*. MBio.

[46] Choe D., Kim U., Hwang S., Seo S.W., Kim D., Cho S. (2023). Revealing causes for false-positive and false-negative calling of gene essentiality in *Escherichia coli* using transposon insertion sequencing. mSystems.

[47] diCenzo G.C., Galardini M., Fondi M. (2021). Tn-core: functionally interpreting transposon-sequencing data with metabolic network analysis. Methods Mol. Biol..

[48] Jang Y.S., Seong H.J., Kwon S.W., Lee Y.S., Im J.A., Lee H.L. (2021). *Clostridium acetobutylicum atpG*-knockdown mutants increase extracellular pH in batch cultures. Front. Bioeng. Biotechnol..

[49] Jensen P.R., Michelsen O. (1992). Carbon and energy metabolism of atp mutants of *Escherichia coli*. J. Bacteriol..

[50] Chaudhuri R.R., Allen A.G., Owen P.J., Shalom G., Stone K., Harrison M. (2009). Comprehensive identification of essential *Staphylococcus aureus* genes using transposon-mediated differential hybridisation (TMDH). BMC Genomics.

[51] Yang Y., Zhu Q., Zhong Y., Cui X., Jiang Z., Wu P. (2020). Synthesis, anti-microbial and anti-inflammatory activities of 18β-glycyrrhetinic acid derivatives. Bioorg. Chem..

[52] Long D.R., Mead J., Hendricks J.M., Hardy M.E., Voyich J.M. (2013). 18β-glycyrrhetinic acid inhibits methicillin-resistant *Staphylococcus aureus* survival and attenuates virulence gene expression. Antimicrob. Agents Chemother..

[53] Marreddy R.K.R., Olaitan A.O., May J.N., Dong M., Hurdle J.G. (2021). Ebselen not only inhibits *Clostridioides difficile* toxins but displays redox-associated cellular killing. Microbiol. Spectr..

[54] Ikuta K., Segawa H., Sasaki S., Hanazaki A., Fujii T., Kushi A. (2018). Effect of Npt2b deletion on intestinal and renal inorganic phosphate (Pi) handling. Clin. Exp. Nephrol..

[55] Lee J.S., Wang R.X., Goldberg M.S., Clifford G.P., Kao D.J., Colgan S.P. (2020). Microbiota-sourced purines support wound healing and mucous barrier function. iScience.

[56] Mishima E., Ichijo M., Kawabe T., Kikuchi K., Akiyama Y., Toyohara T. (2020). Germ-free conditions modulate host purine metabolism, exacerbating adenine-induced kidney damage. Toxins (Basel).

[57] Putluri N., Shojaie A., Vasu V.T., Vareed S.K., Nalluri S., Putluri V. (2011). Metabolomic profiling reveals potential markers and bioprocesses altered in bladder cancer progression. Cancer Res..

[58] Kechin A., Boyarskikh U., Kel A., Filipenko M. (2017). Cutprimers: a new tool for accurate cutting of primers from reads of targeted next generation sequencing. J. Comput. Biol..

[59] Ewels P., Magnusson M., Lundin S., Kaller M. (2016). Multiqc: summarize analysis results for multiple tools and samples in a single report. Bioinformatics.

[60] Dobin A., Davis C.A., Schlesinger F., Drenkow J., Zaleski C., Jha S. (2013). Star: ultrafast universal RNA-seq aligner. Bioinformatics.

[61] Liao Y., Smyth G.K., Shi W. (2014). Featurecounts: an efficient general purpose program for assigning sequence reads to genomic features. Bioinformatics.

[62] Love M.I., Huber W., Anders S. (2014). Moderated estimation of fold change and dispersion for RNA-seq data with deseq2. Genome Biol..

[63] Babakhani F., Bouillaut L., Gomez A., Sears P., Nguyen L., Sonenshein A.L. (2012). Fidaxomicin inhibits spore production in *Clostridium difficile*. Clin. Infect. Dis..

[64] Zhou B., Yuan Y., Shi L., Hu S., Wang D., Yang Y. (2021). Creation of an anti-inflammatory, leptin-dependent anti-obesity celastrol mimic with better druggability. Front. Pharmacol..

[65] Zhou F., Hamza T., Fleur A.S., Zhang Y., Yu H., Chen K. (2018). Mice with inflammatory bowel disease are susceptible to *Clostridium difficile* infection with severe disease outcomes. Inflamm. Bowel Dis..

[66] Chen X., Katchar K., Goldsmith J.D., Nanthakumar N., Cheknis A., Gerding D.N. (2008). A mouse model of *Clostridium difficile*-associated disease. Gastroenterology.

[67] Babcock G.J., Broering T.J., Hernandez H.J., Mandell R.B., Donahue K., Boatright N. (2006). Human monoclonal antibodies directed against toxins A and B prevent *Clostridium difficile*-induced mortality in hamsters. Infect. Immun..

[68] Dzunkova M., D'Auria G., Xu H., Huang J., Duan Y., Moya A. (2016). The monoclonal antitoxin antibodies (actoxumab-bezlotoxumab) treatment facilitates normalization of the gut microbiota of mice with *Clostridium difficile* infection. Front. Cell. Infect. Microbiol..

[69] Bender K.O., Garland M., Ferreyra J.A., Hryckowian A.J., Child M.A., Puri A.W. (2015). A small-molecule antivirulence agent for treating *Clostridium difficile* infection. Sci. Transl. Med..

[70] Tam J., Hamza T., Ma B., Chen K., Beilhartz G.L., Ravel J. (2018). Host-targeted niclosamide inhibits *C. difficile* virulence and prevents disease in mice without disrupting the gut microbiota. Nat. Commun..

[71] Warren C.A., van Opstal E.J., Riggins M.S., Li Y., Moore J.H., Kolling G.L. (2013). Vancomycin treatment's association with delayed intestinal tissue injury, clostridial overgrowth, and recurrence of *Clostridium difficile* infection in mice. Antimicrob. Agents Chemother..

[72] Cosmetic Ingredient Review Expert Panel (2007). Final report on the safety assessment of glycyrrhetinic acid, potassium glycyrrhetinate, disodium succinoyl glycyrrhetinate, glyceryl glycyrrhetinate, glycyrrhetinyl stearate, stearyl glycyrrhetinate, glycyrrhizic acid, ammonium glycyrrhizate, dipotassium glycyrrhizate, disodium glycyrrhizate, trisodium glycyrrhizate, methyl glycyrrhizate, and potassium glycyrrhizinate. Int. J. Toxicol..

