# Supplementary material for: Chemical genetic analysis of enoxolone inhibition of Clostridioides difficile toxin production reveals adenine deaminase and ATP synthase as antivirulence targets
Source: J Biol Chem. 2024 Sep 27;300(11):107839. doi: 10.1016/j.jbc.2024.107839 (PMC11566853; doi:10.1016/j.jbc.2024.107839)
Supplement: Supporting Information [file mmc1.pdf]

Supporting Information for

Chemical genetic analysis of enoxolone inhibition of *C. difficile* toxin production reveals adenine deaminase and ATP synthase as anti-virulence targets

Ravi K. R. Marreddy[1], Gregory A. Phelps[2,3], Kelly Churion[1], Jonathan Picker[1], Reid Powell[4], Philip T. Cherian[2], John J. Bowling[2], Clifford C. Stephan[4], Richard E. Lee[2], Julian G. Hurdle[1#]

[1]Center for Infectious and Inflammatory Diseases, Institute of Biosciences and Technology, Texas A&M Health Science Center, Houston, Texas, USA

[2]Department of Chemical Biology and Therapeutics, St. Jude Children's Research Hospital, Memphis, TN, USA

[3]Graduate School of Biomedical Sciences, St. Jude Children's Research Hospital, Memphis TN, 38103 USA

[4]Center for Translational Cancer Research, Institute of Biosciences and Technology, Texas A&M Health Science Center, Houston, Texas, USA

G.A.P and K.C. equally contributed to this work.

[#]Corresponding author Email: jhurdle@tamu.edu

Index of Supporting Information	
**Supplementary Figure 1**	Glycyrrhizin does not inhibit toxin production.
**Supplementary Figure 2**	Enoxolone (ENX) inhibits toxin production in various *C. difficile* ribotype and strains
**Supplementary Figure 3**	Identification of molecular target(s) for enoxolone (ENX)
**Supplementary Figure 4**	Characterization of antisense knockdown constructs.
**Supplementary Figure 5**	Molecular binding analysis by Surface Plasmon Resonance (SPR) and Isothermal Titration Calorimetry (ITC)
**Supplementary Figure 6**	Transcriptional response for *C. difficile* R20291 to enoxolone.
**Supplementary Figure 7**	Growth kinetics for *C. difficile* R20291 under varying conditions
**Supplementary Figure 8**	Experiments to test the efficacy of enoxolone (ENX) in mice with CDI colitis
**Supplementary Table 1**	Identification of proteins targeted by enoxolone using affinity-based proteomics
**The below are separately uploaded supporting information**	
**Supplementary Table 2**	List of proteins identified through affinity-based proteomics
**Supplementary Table 3**	Significantly transcribed genes in *C. difficile* R20291 exposed to enoxolone 16 μM

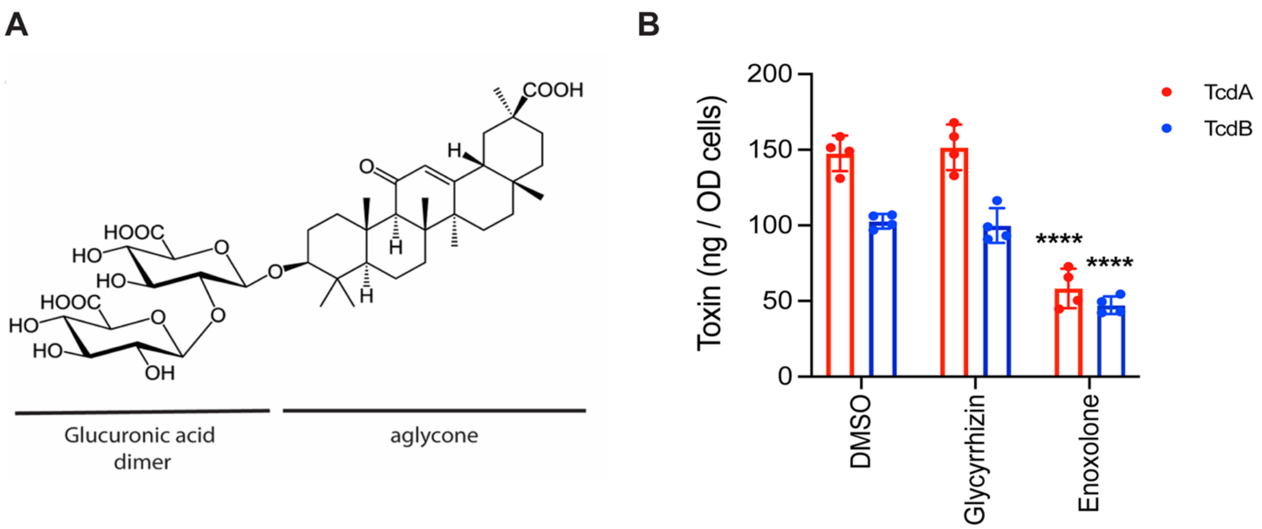

Figure S1: Glycyrrhizin does not inhibit toxin production. (A) The structure of glycyrrhizin consists of a glucuronic acid dimer linked to enoxolone by a glycosidic bond. **(B)** *C. difficile* R20291 grown to exponential phase (OD_{600}nm≈ 0.3 were exposed to 100 μM glycyrrhizin or enoxolone; toxin levels for TcdA (red bars) and TcdB (blue bars) in culture supernatants were quantified. Data was from three biological replicates and shown as mean ± SEM (one-way ANOVA with Tukey's test; **** P<0.0001; done using Graphpad prism version 9.3.1).

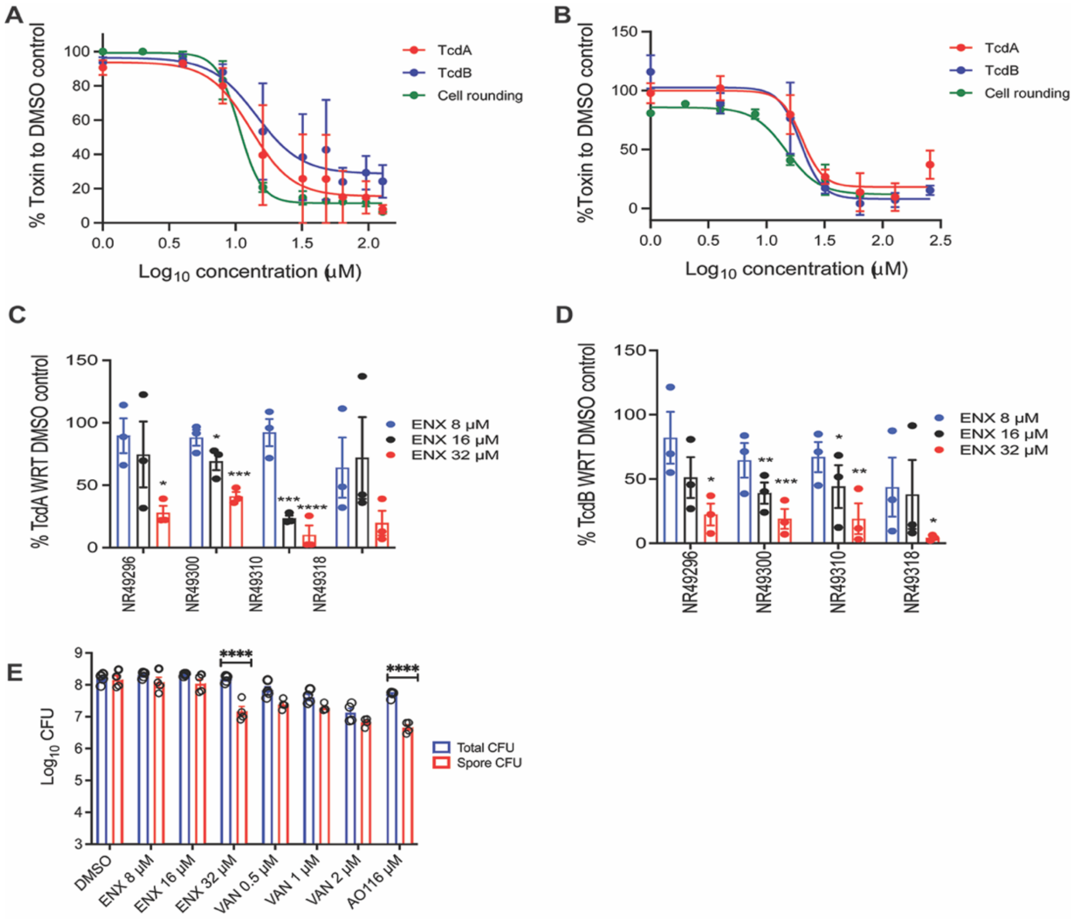

Figure S2: Enoxolone (ENX) inhibits toxin production in various *C. difficile* ribotype and strains. (A & B) Enoxolone dose response curves for toxin levels against *C. difficile* ribotype (RT) 027 strains CD196 (A) and UK1 (B). After exposing exponential (OD_{600} ≈ 0.3) cells to various concentrations of ENX for 24 h, toxins were quantified by ELISA for TcdA (red) and TcdB (blue) and by cytopathic cell rounding (green; CR). The EC_{50} observed for different RT027 strains through various methods are: CD196 (TcdA 13.01 μM, [Hill slope=-2.761]; TcdB 14.37 μM, [Hill slope=-2.570]; CR 10.66 μM, [Hill slope=-5.183]); and UK1 (TcdA 20.23 μM, [Hill slope=-4.772]; TcdB 19.68 μM, [Hill slope=-4.649]; CR 14.81 μM, [Hill slope=-3.083]). See Figures 1 and 2 in manuscript for comparison of above EC_{50}s and Hill slopes for R20291 (i.e., EC_{50}s TcdA 11.38 μM; TcdB 14.29 μM; CR 7.77 μM). The EC_{50}s

and negative Hill slopes are indicative of downhill inhibition curves, where increases in drug concentration caused a decrease in toxin production with a narrow threshold between effective and ineffective drug concentrations, suggesting a complex mode of action. **(C & D)** Effect of enoxolone on TcdA (**C**) and TcdB (**D**) production in varying *C. difficile* ribotype strains i.e., NR49296 (RT-014), NR49300 (RT-020), NR49310 (RT-078) and NR49318 (RT-106). Cultures were exposed to enoxolone at 8, 16 and 32 µM (based on the adopted EC_{50} against R20291 of 16 µM, which did not substantially affect growth). Toxin levels are relative to DMSO control. Data was obtained from three biological replicates and are presented as mean ± SEM (one-way ANOVA with Tukey's test; * P<0.05, ** P<0.01, *** P<0.001; done using Graphpad prism version 9.3.1). (**E**) Raw viable counts of enoxolone inhibition of sporulation; cells (OD_{600} ≈ 0.3) were treated with ENX, vancomycin (VAN) or acridine orange (AO) and total viable and spores enumerated five days and data used to calculate the % of heat resistant spores in Figure 2D of the main text. The CFUs (colony forming units) are per ml; n = 4 biological replicates. Using Graphpad prism version 9.3.1, statistical analysis on total viable counts versus spore counts for each test condition was by one-way ANOVA with Tukey's test; ****, P<0.0001.

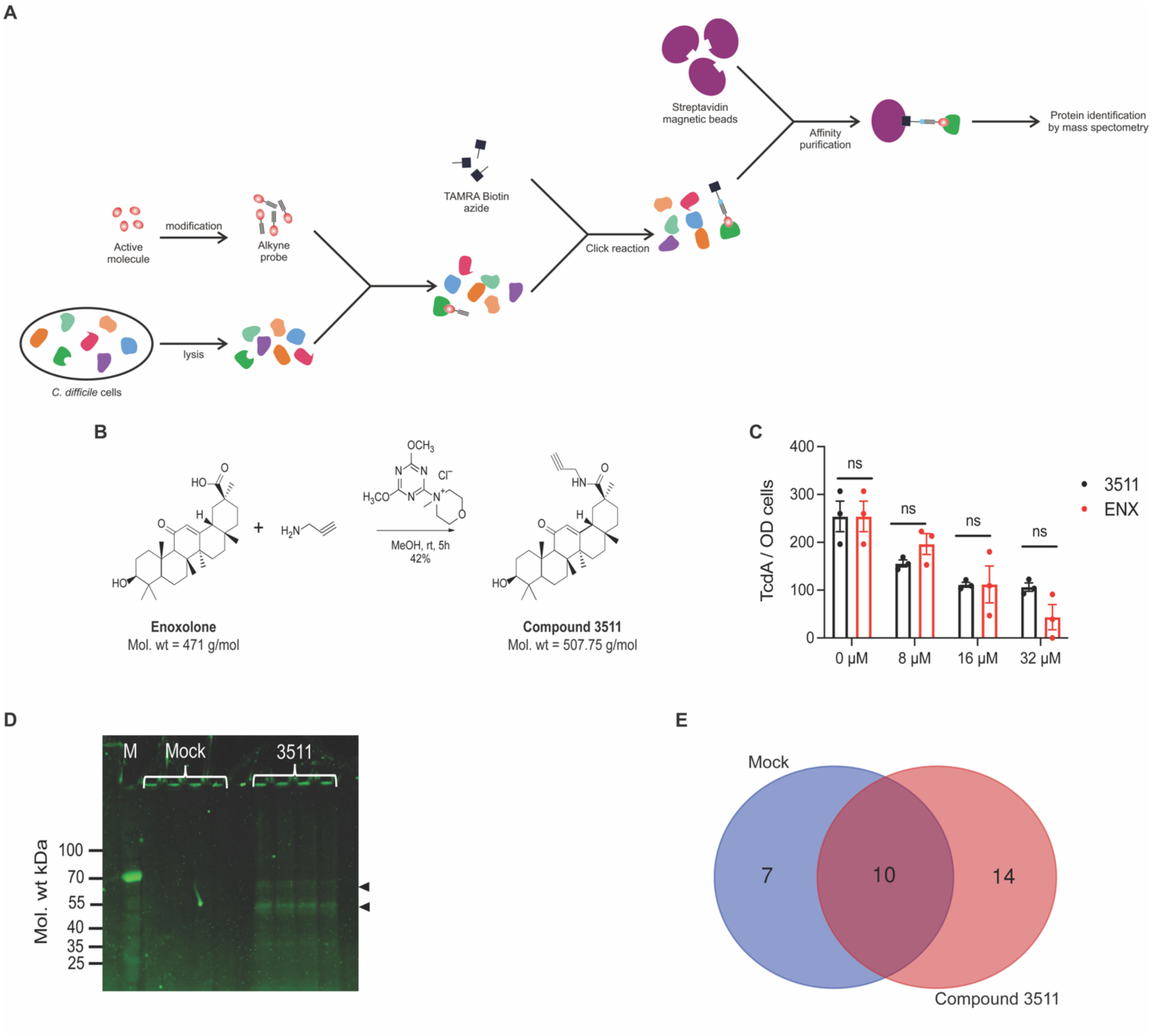

Figure S3: Identification of molecular target(s) for enoxolone (ENX). (A) Schematic of the click chemistry pull-down for target identification. Compound 3511 was coupled to carboxymethylrhodamine (TAMRA) azide through CuAAC click reaction followed by incubation with cell lysates. Proteins bound to the streptavidin-conjugated TAMRA azide were immobilized on streptavidin magnetic beads and the enriched proteins were identified by mass spectrometry analysis. **(B)** Synthetic scheme of compound 3511. The amine group from propargylamine was coupled to enoxolone in presence of 4-(4,6-dimethoxy-1,3,5-

triazin-2-yl)-4-methylmorpholinium chloride (DMT-MM) in methanol at room temperature. **(C)** Quantification of TcdA from culture supernatants of R20291 exposed to equivalent concentrations of enoxolone (red bars) or compound 3511 (black bars) for 24 h. Data from three biological replicates, shown as mean ± SEM, indicate inhibition by ENX and 3511 were not statistically different (based on one-way ANOVA with Tukey’s test in Graphpad prism version 9.3.1). **(D)** In-gel fluorescence detection for proteins bound to TAMRA biotin azide after click reaction followed by affinity purification with streptavidin magnetic beads; lanes labelled Mock (enoxolone) or 3511 show proteins derived from membrane fractions (cytosolic fractions showed no in-gel fluorescence [*data not shown*]); technical aliquots from the mock or 3511 samples were ran on the gel. **(E)** Venn diagram showing overlapping and unique proteins identified from enoxolone mock and click reactions. The Venn diagram was generated through bioinformatics tool at VIB / UGent Bioinformatics & Evolutionary Genomics.

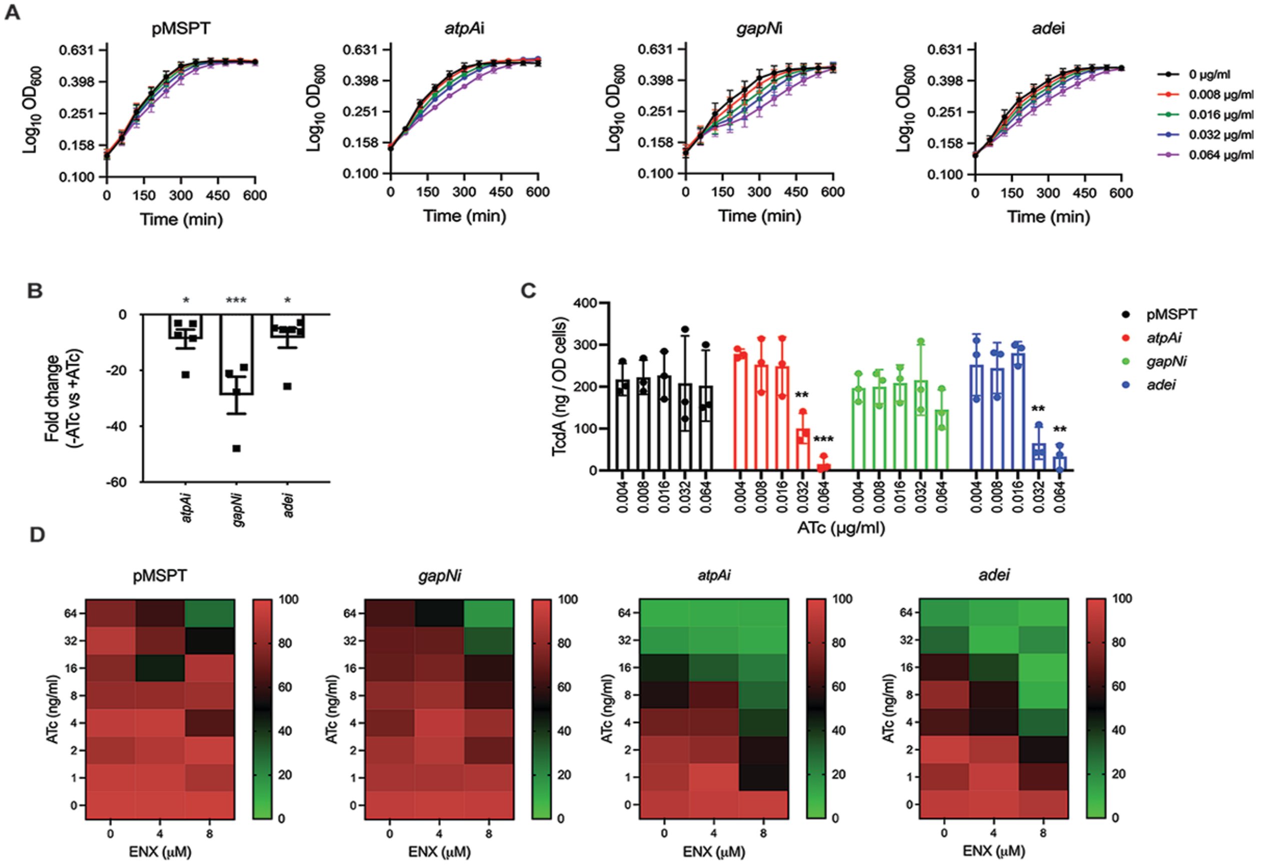

Figure S4: Characterization of antisense knockdown constructs. (A) Growth kinetics for the knockdown strains analyzed in a 96-well plate. Growth was analyzed for *C. difficile* R20291 harboring respective plasmids in different concentration of ATc (µg/ml) i.e., 0 (black), 0.008 (red), 0.016 (green), 0.032 (blue) and purple (0.064). Data was from four biological replicates. **(B)** mRNA levels for the respective gene knockdowns i.e., *atpA* (red bar), *gapN* (green bar) and *ade* (blue bar) were analyzed by RT-qPCR. Cells were grown to OD_{600} ≈ 0.3 and the antisense RNA expression was induced with 0.032 µg/ml of ATc for 1 h. Fold changes were calculated as the difference of mRNA between untreated cells and those exposed to ATc (0.032 µg/ml). Data was from three biological replicates and statistical significance was assessed from ΔCt values, comparing untreated and with samples treated

with ATc (0.032 µg/ml), by one-way ANOVA with Tukey's test: * P<0.05, *** P<0.001 in Graphpad prism 9.3.1. **(C)** TcdA protein in R20291 with respective antisense knockdowns i.e., pMSPT (black), *atpA*i (red), *gapN*i (green) and *ade*i (blue) were cultured in different concentrations of ATc. After 24 h, TcdA was quantified in the culture supernatants. Data was from three biological replicates and shown as mean ± SEM (one-way ANOVA with Tukey's test; **, *P*<0.01; ***, *P*<0.001; done using Graphpad prism version 9.3.1). **(D)** Potentiation of enoxolone inhibition of toxin production by gene silencing. Cells carrying pMSPT, *gapN*i, *atpA*i and *ade*i were exposed to varying ATc concentrations (y-axis) with and without varying sub-inhibitory concentrations of enoxolone (x-axis), for 24 h. Effect on toxin production was then evaluated in cell rounding assays. The data is shown as heat maps plotted in Graphpad prism version 9.3.1, demonstrating that *atpA*i and *ade*i were synergistic with enoxolone.

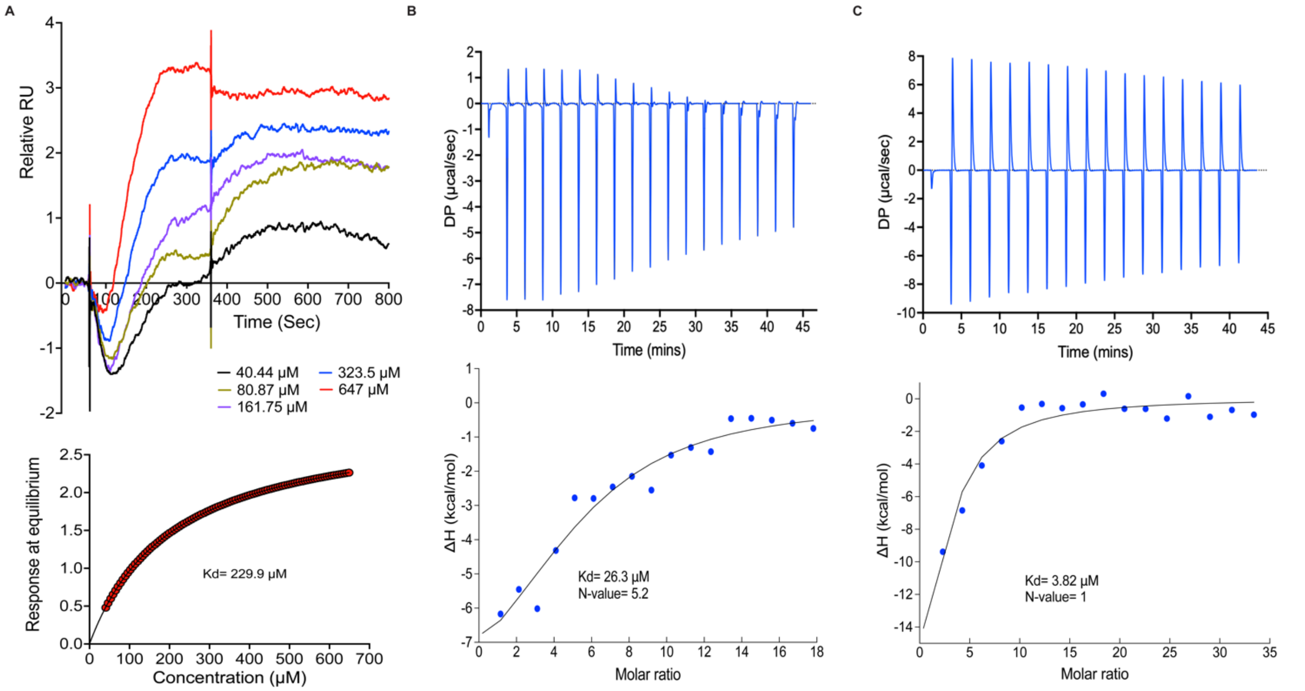

Figure S5: Molecular binding analysis by Surface Plasmon Resonance (SPR) and Isothermal Titration Calorimetry (ITC). (A) 6-Chloropurine shows weak binding to *C. difficile* adenine deaminase by SPR; top panel shows dose response representative sensogram and bottom panel shows corresponding response at equilibrium (K_d=264 ± 42.90 µM of 4 replicates). **(B, C)** Binding isotherms for the interaction of enoxolone (B) and adenine (C) were generated by titrating the compounds into an ITC cell with adenine deaminase (10 or 1 µM, respectively for the compounds). Top panels depict heat differences upon injection of compounds and the bottom panel shows molar ratio (ligand/protein) versus enthalpy changes. Data were fitted to a one site binding model and the binding affinities calculated using Origin software were: enoxolone K_d = 19.94 ± 9.74 µM of 4 replicates (N-value= 5.22 ± 3.54) and adenine K_d = 5.28 ± 2.07 of 2 replicates (N-value= of 2.32 ± 1.86). We speculate enoxolone's N-value indicates it makes multiple

binding interactions with Ade. The lower N-value of adenine suggests that it engages in more specific binding, with the catalytic site(s) of Ade. Results above are shown as mean ± standard deviation.

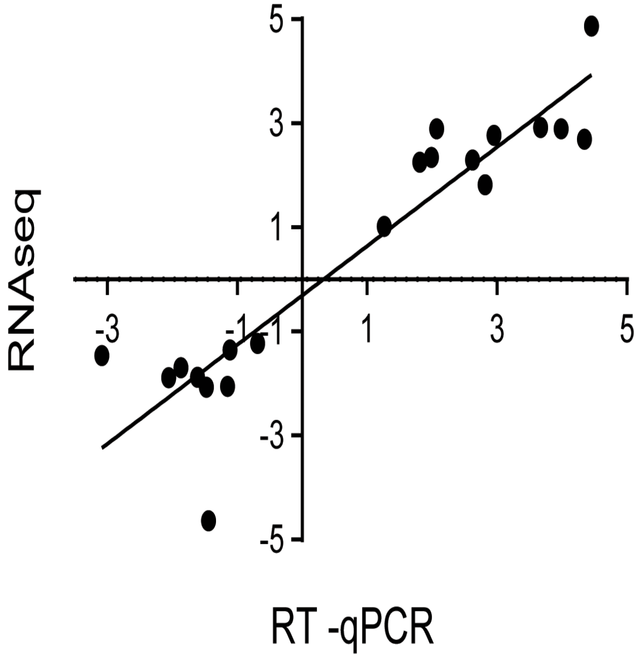

Figure S6: Validation of transcriptional response for *C. difficile* R20291 to enoxolone. Gene expression was analyzed by quantifying mRNA in R20291 (OD_{600} ≈ 0.3) exposed to 16 µM (1 x EC_{50}) enoxolone for 30 min. Pearson correlation plot for a panel of 20 genes (11 – upregulated and 9 – down regulated) representing expression fold changes between RNAseq and RT-qPCR; R^2 was 0.8499 (P<0.0001) in Graphpad prism 9.5.1. Log_2 fold change values are the calculated difference in mRNA between cells exposed to DMSO and 16 µM enoxolone.

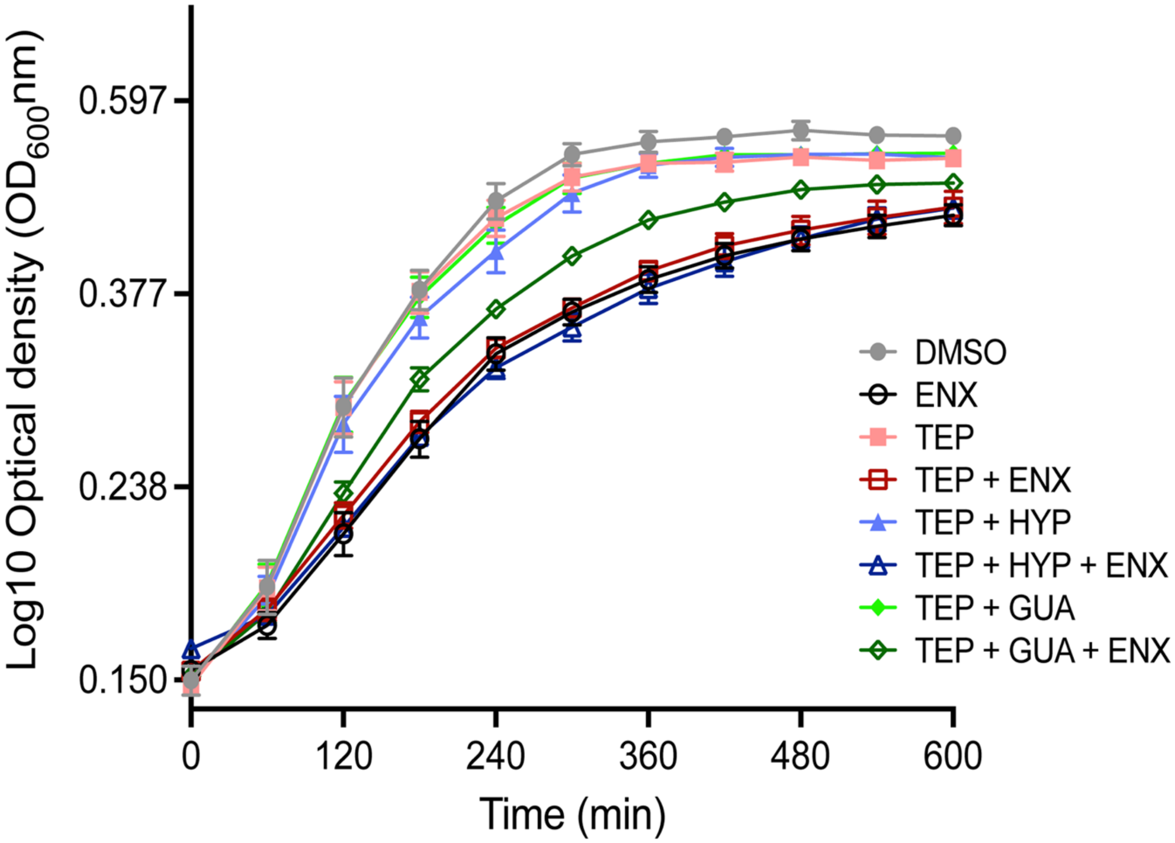

Figure S7: Growth kinetics (n = 3) for *C. difficile* R20291 under varying conditions. Triethyl phosphate (TEP) supplementation did not restore growth in enoxolone (ENX), while unlike guanine (GUA) when added to cells exposed to enoxolone. Cells were exposed to 1% (v/v) DMSO or 32 µM enoxolone in the presence or absence of 250 µM TEP and/or 250 µM hypoxanthine (HYP) or 250 µM guanine. Growth kinetics were analyzed in a 96-well plate by reading absorbance at 600 nm for every 1 h for a span of 10 hrs.

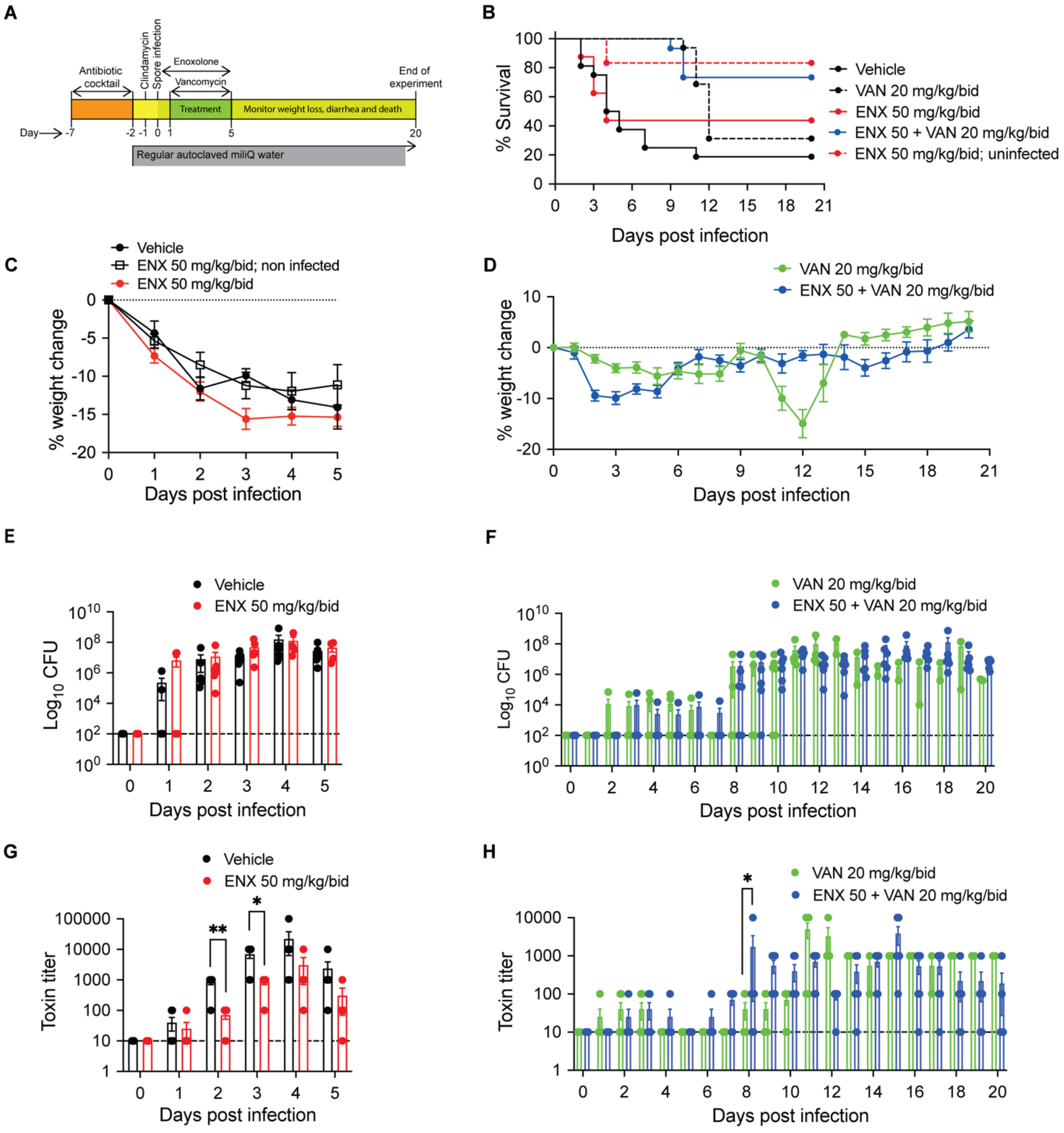

Figure S8: Efficacy of enoxolone (ENX) alone or in combination with vancomycin in mice with CDI colitis. (**A**) Schematic representation of the CDI colitis model. There were four groups of infected mice (16 males and 16 females in each group) as follows: vehicle (10% DMSO in corn oil), ENX (50 mg/kg), vancomycin (VAN, 20 mg/kg) and combination

(ENX 50 mg/kg and VAN 20 mg/kg). An uninfected control (n=6) was treated with ENX. Mice were treated twice daily (bid) via oral gavage. (**B**) Kaplan-Meier plot of animal survival. Log-rank (Mantel-Cox) statistical analysis of survival of infected mice showed ENX, VAN and ENX + VAN combination had P-values of 0.8140 (not significant), 0.0055 and 0.0003 respectively, compared to the vehicle. Survival rates were 43.75% and 18.75%, for ENX and vehicle respectively. Mice given VAN monotherapy had a survival rate of 31.5% versus 73.4% for mice given ENX/VAN. (**C and D**) Percent weight change, with respect to day 0, before infection; **C** shows infected mice given vehicle or ENX compared to uninfected mice receiving only ENX; **D** shows infected mice given VAN or ENX + VAN. In **C**, uninfected mice given ENX experienced significant weight loss; hence, weight loss is greater in mice infected with *C. difficile* and given ENX in **C**. More pronounced weight loss is also seen in infected mice treated with ENX + VAN when compared to mice given VAN alone in **D**. Weight loss caused by ENX in mice may be due to its suppression of food-intake in a leptin-dependent manner (1). (**E and F**) Analysis of *in vivo C. difficile* colonization (bioburden in feces) of mice treated with vehicle or ENX is in **E** and that for VAN or ENX + VAN is in **F**; the limit of detection was 2 $\log_{10}$ CFU/g. There was no significant difference in spore bioburdens in E, but these burdens were lower in the animal groups in F that were given VAN. (**G and H**) Toxin titers in feces, as determined by cell rounding against Vero cells, using feces from mice treated with vehicle or ENX in **G**; and VAN or ENX + VAN in **H**; the dashed line indicates limit of detection of 10^1 toxin titer. In G and H, head-to-head statistical analysis of toxin titers on specific days in the two groups were done by two-tailed multiple unpaired t-test with correction for multiple comparisons using the Holm-Šídák method and alpha set to 0.05; * P<0.05 and ** P<0.01. In panels C, E and G animal numbers for vehicle and ENX treatment groups at days 4 were 11 and 10 mice, respectively. Fecal toxin titers

of ENX treated mice were ~1 log less than mice given the vehicle in G, suggesting ENX monotherapy reduced toxins *in vivo*, but ENX also accelerated weight loss of infected mice leading to an overall negative outcome. ENX is known to have anti-inflammatory properties, including reducing synthesis of TNF-alpha and interleukin-6 (2, 3). This factor along with lower bioburdens caused by VAN might explain why the ENX + VAN combination group had better survival, although microbiota changes cannot be ruled out. In **H**, during days 1-5, animals had toxin titers that were close to the detection limit. This was related to lower bioburdens driven by the antibiotic VAN (i.e., when comparing vehicle to VAN and ENX to VAN/ENX, the CFUs were lower by 2.84-4.06 and 3.68-5.07 Logs, respectively).

Methodology for CDI colitis mouse model. Studies were conducted under an animal use protocol approved by The Institutional Animal Care and Use Committee of Texas A&M University. The CDI colitis mouse model was adapted from a previously reported model (4), by using a lower concentration of dextran sodium sulphate (DSS, 1% w/v instead of 3% w/v) and administering an antibiotic cocktail and DSS for 5 days instead of 3 days. Antibiotics were kanamycin (0.4 mg/ml), metronidazole (0.215 mg/ml), vancomycin (0.045 mg/ml), gentamicin (0.035 mg/ml) and colistin (850 U/ml) (5). C57BL/6 mice (6 weeks), from Envigo, were treated with the antibiotic cocktail and DSS with 1% (w/v) for 5 days and 2 days later mice were given clindamycin (10 mg/kg via i.p.). About 20 h later, mice were challenged with gavaged 10^5 viable spores of R20291. Oral gavage of enoxolone (50 mg/kg) or vehicle (10% DMSO in corn oil) was started ~2 h after infection, whereas vancomycin (20 mg/kg) treatment was started at 24 h; respectively, this reflects prophylaxis with an antivirulence agent and treatment with an antimicrobial; mice were dosed twice daily. CDI typical develops within 20 h in mice. Since enoxolone is predominantly a non-

antibiotic, early dosing was carried out, which is comparable to bezlotoxumab (6, 7), ebselen (8) and niclosamide (9). Vancomycin was dosed a day after infection (10). The dose of enoxolone was below its oral LD50 of >610 mg/kg in mice, but above the average daily intake of 100 mg for glycyrrhizin in humans, as recommended by the European Union (11). Mice were monitored thrice daily after infection for signs of morbidity (decreases in weight and temperature, and changes in appearance [wet tail, hunched posture, ruffled coat) and those that became moribund were euthanized. During the experiment, fecal samples were collected and stored at -80°C until use. CFUs were enumerated per gram of fecal pellet in 1 ml of sterile PBS. After heating at 65°C for 30 min, serial dilutions plated on selective and differential media for *C. difficile* (cycloserine-cefoxitin fructose agar with 0.1% (w/v) taurocholate). Toxin tires were quantified from the fecal pellets by cytopathic cell rounding assay.

Table-S1: Screened phytochemicals/metabolites in this study

Compound name	Mol. Wt.	Compound class	Source	Structure
Sclareolide	250.8	Sesquiterpene lactone	Food additive and flavoring	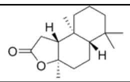
Schisantherin A	536.57	Dibenzocyclooctadiene lignan	Found in traditional Chinese medicine from fruit of *Schisandra sphenanthera*	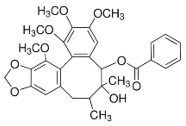
Sarsasapogenin	416.64	Steroidal sapogenin (triterpenoid)	Found in Chinese medicinal herb *Anemarrhena asphodeloides bunge*	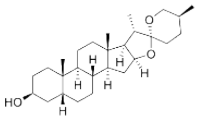
Sclareol	308.5	Bicyclic diterpene alcohol	A main component *Salivia sclarea* essential oil used in flavoring food and beverages, cosmetic products, and traditional medicine	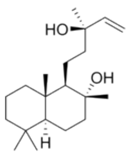
Leucomisine (Prestw-NAT-0234)	246.3	Sesquiterpene lactone	Found in *Artemisia ludoviciana*	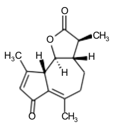
Proxyphylline (Prestw-NAT-0214)	238.25	Methylxanthines	Proxyphylline is a methylxanthine and synthetic derivative of the natural product theophylline, found in green and black tea.	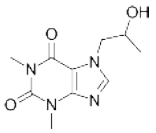
Strophantine octahydrate (Prestw-NAT-0107)	728.79	Glycoside	*Strophanthus gratus* and other plants of the Apocynaceae	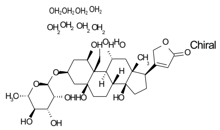

Ipratropium bromide (Prestw-NAT-0130)	412.37	Tropane alkaloid	Chemical analog of natural product atropine used as a bronchodilator	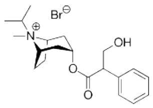
Monocrotaline (Prestw-NAT-0152)	325.36	Pyrrolizidine alkaloid	In plants from genus Crotalaria	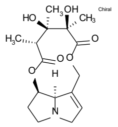
Vulpinic acid (Prestw-NAT-0344)	322.32	Furan	Isolated from Lichen species (e.g., *Letharia vulpina*)	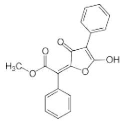
Kinetin riboside (Prestw-NAT-0061)	347.33	6-Furfurylaminopurine riboside	A cytokinin riboside plant hormone, also found in coconut water	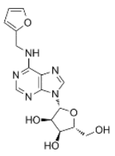
Verbenalin (Prestw-NAT-0281)	388.37	Iridoid glycoside	Found in *Verbena officinalis*, a plant used in traditional Chinese medicine; considered to be anti-inflammatory	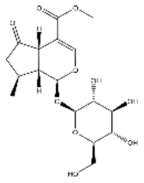
Bakankosine (Prestw-NAT-0255)	357.36	Monoterpene alkaloid		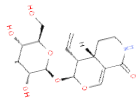
7-glucosylombelliferone	324.29	Coumarine		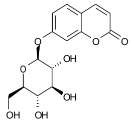
18β-glycyrrhetinic acid (enoxolone; Prest-NAT-310)	470.7	Pentacyclic triterpenoid	Derived from glycyrrhizic acid (GRA) hydrolysis. GRA is from licorice root (*Glycyrrhiza glabra*)	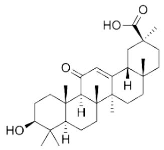
Indirubin	262.27	Indole	Roots and leaves of *Indigofera tinctoria* (Indiga plant)	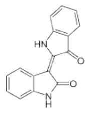

Cynarin	516.47	Hydroxycinnamic acid	*Synara cardunculus* (Artichoke)	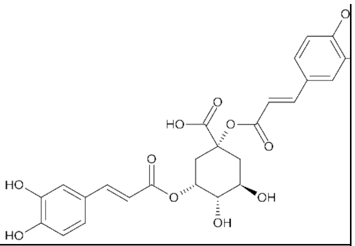
Austricine hydrate	280.32	Sesquiterpene		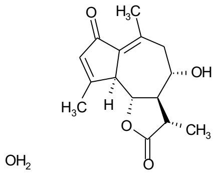
Solasodine	413.65	Steroid	Solanaceae family	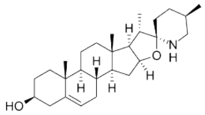
Harpagoside	494.5	Iridoid glycoside	*Harpagophytum procumbens*	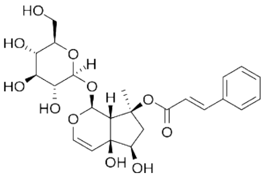
β-sistosterol	424.72	Phytosterol	Vegetable oil, nuts, avacados and salad dressings	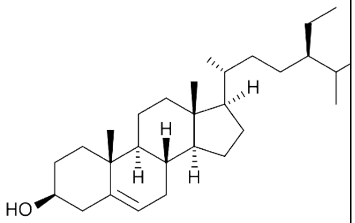
α-santonin	246.31	Lactone	*Artemisia maritima*	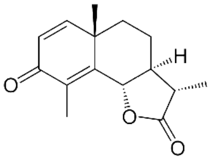
Leucomisine	246.31	Sesquiterpene lactone	*Artemisia* Sps. and *Achillea* Sps.	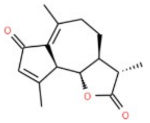

Trimethylcolchicinic acid	343.38	Colchicine		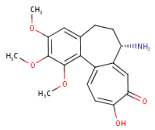
Ajmaline	326.44	Alkaloid	*Rauvolfia serpentina*	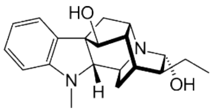
Colchicine	399.45	Alkaloid	*Colchicum sps*.	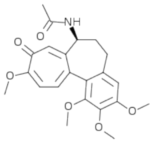
Pilocarpine nitrate	271.28	Alkaloid		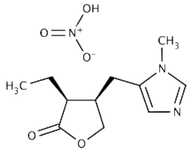
Thiocolchicoside	563.63	Alkaloid	*Gloriosa superba*	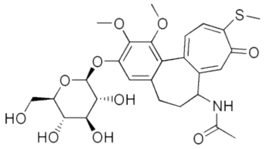
Kainic acid	213.24	Amino acid	Seaweed	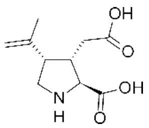
Sedanolide	194.3	Benzofuran	In coriander, green vegetables and celery oil	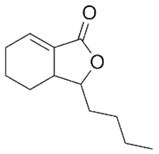
Cafestol	316.4	Diterpenoid	Seeds of *Coffea arabica*	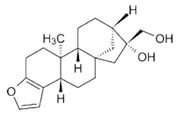
Kahweol	314.4	Diterpenoid	Seeds of *Coffea arabica*	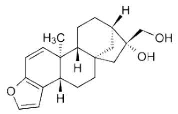

Ginkgolide B	424.4	Terpenic lactone	*Ginkgo biloba*	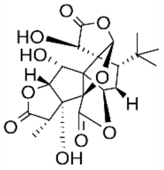
Bilobalide	326.3	Sesquiterpene trilactone	*Ginkgo biloba*	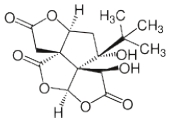
parthenolide	248.3	Sesquiterpene lactone	*Tanacetum parthenium*	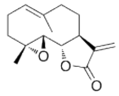
Rauwolscine	390.9	Alkaloid	*Rauwolfia* and *Pausinystalia Sps*.	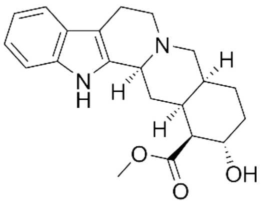
6-gingerol	294.4	Polyphenol	*Zingiber officinale* (ginger)	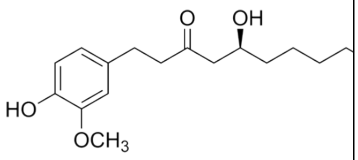
Kenpaullone	327.2	Competitive inhibitor of cyclin-dependent kinases (CDKs)		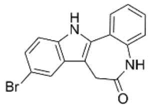
1-azakenpaullone	328.2	Tau-protein kinase inhibitor		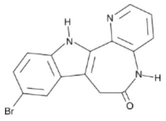
Tomatidine	452.1	Steroidal alkaloid	Skin and leaves of *Solanum lycopersicum* (tomatoes)	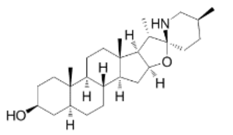

Bufalin	386.5	Cardiotonic steroid	Toad venom	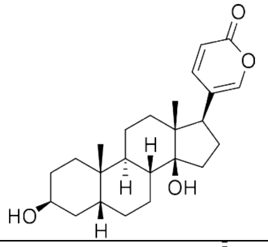
Ursolic acid	456.7	Pentacyclic triterpenoid	*Mirabilis Jalapa* and in many fruits and herbs used in daily life like apples, basil, cranberries	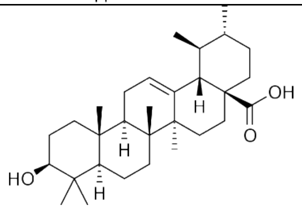
Abscisic acid	264.3	Plant hormone	All plants and some plant pathogenic fungi	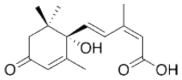
Galantamine	287.4	Alkanoid	In *Galanthus Sps*. and *Amaryllidaceae sps*.	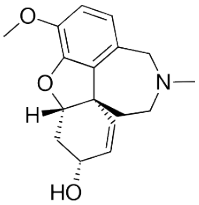
Celastrol	450.6	Triterpine	Root extracts of *Tripterygium wilfordi* (thunder god vine)	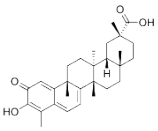
Ursodeoxycholic acid	414.6	Bile acid	Bile acid	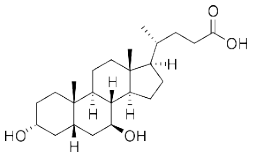
Cryptotanshinone	296.4	Diterpenoid quinones	Roots of *Salvia miltiorrhiza*	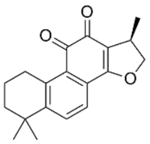
Tanshinone IIA	294.3	Diterpenoid	*Salvia miltiorrhiza*	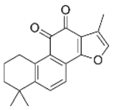

24 alpha-ethylcholesterol	414.7	Phytosterol	Vegetable oils	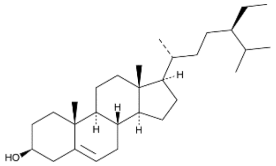
Tannic acid	1701.19	Polyphenol	*Caesalpinia spinosa*, *Rhus semialata, Quercus infectoria, Rhus coriaria*	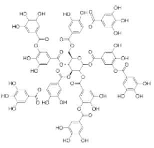
Urolithin A	228.2	Benzo-coumarins	Metabolic byproduct of ellagitannis	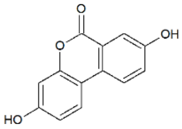
Urolithin B	212.21	Phenolic acompound	Metabolic byproduct of ellagitannis	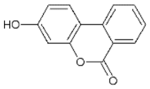
Kyneuric acid	189.16	Quinolinemonocarboxylic acid	Metabolic byproduct of L-tryptophan	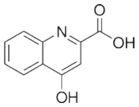
Pyrogallol	126.11	Benzenetriols	*Myriophyllum spicatum*	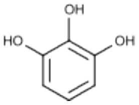
Enterodiol	302.4	Lignan	Metabolic byproduct from plant lignan in intestine by bacteria	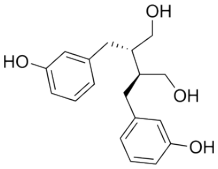
Epicatechin	290.27	Flavanols	Abundantly present in cacao and cacao products	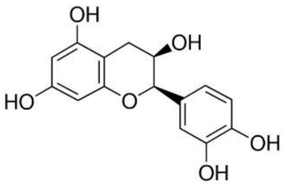

Enterolactone	298.34	Lignan	Metabolic byproduct of plant lignan in intestine by bacteria	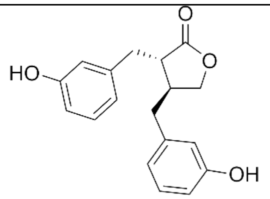

References

1. Zhou, B., Yuan, Y., Shi, L., Hu, S., Wang, D., Yang, Y. *et al.* (2021) Creation of an Anti-Inflammatory, Leptin-Dependent Anti-Obesity Celastrol Mimic with Better Druggability Front Pharmacol **12**, 705252 10.3389/fphar.2021.705252
2. Yang, Y., Zhu, Q., Zhong, Y., Cui, X., Jiang, Z., Wu, P. *et al.* (2020) Synthesis, anti-microbial and anti-inflammatory activities of 18beta-glycyrrhetinic acid derivatives Bioorg Chem **101**, 103985 10.1016/j.bioorg.2020.103985
3. Long, D. R., Mead, J., Hendricks, J. M., Hardy, M. E., andVoyich, J. M. (2013) 18beta-Glycyrrhetinic acid inhibits methicillin-resistant Staphylococcus aureus survival and attenuates virulence gene expression Antimicrob Agents Chemother **57**, 241-247 10.1128/AAC.01023-12
4. Zhou, F., Hamza, T., Fleur, A. S., Zhang, Y., Yu, H., Chen, K. *et al.* (2018) Mice with Inflammatory Bowel Disease are Susceptible to Clostridium difficile Infection With Severe Disease Outcomes Inflamm Bowel Dis **24**, 573-582 10.1093/ibd/izx059
5. Chen, X., Katchar, K., Goldsmith, J. D., Nanthakumar, N., Cheknis, A., Gerding, D. N., andKelly, C. P. (2008) A mouse model of Clostridium difficile-associated disease Gastroenterology **135**, 1984-1992 10.1053/j.gastro.2008.09.002
6. Babcock, G. J., Broering, T. J., Hernandez, H. J., Mandell, R. B., Donahue, K., Boatright, N. *et al.* (2006) Human monoclonal antibodies directed against toxins A and B prevent Clostridium difficile-induced mortality in hamsters Infect Immun **74**, 6339-6347 10.1128/IAI.00982-06
7. Dzunkova, M., D'Auria, G., Xu, H., Huang, J., Duan, Y., Moya, A. *et al.* (2016) The Monoclonal Antitoxin Antibodies (Actoxumab-Bezlotoxumab) Treatment

Facilitates Normalization of the Gut Microbiota of Mice with Clostridium difficile Infection Front Cell Infect Microbiol **6**, 119 10.3389/fcimb.2016.00119

8. Bender, K. O., Garland, M., Ferreyra, J. A., Hryckowian, A. J., Child, M. A., Puri, A. W. *et al.* (2015) A small-molecule antivirulence agent for treating Clostridium difficile infection Sci Transl Med **7**, 306ra148 10.1126/scitranslmed.aac9103
9. Tam, J., Hamza, T., Ma, B., Chen, K., Beilhartz, G. L., Ravel, J. *et al.* (2018) Host-targeted niclosamide inhibits C. difficile virulence and prevents disease in mice without disrupting the gut microbiota Nat Commun **9**, 5233 10.1038/s41467-018-07705-w
10. Warren, C. A., van Opstal, E. J., Riggins, M. S., Li, Y., Moore, J. H., Kolling, G. L. *et al.* (2013) Vancomycin treatment's association with delayed intestinal tissue injury, clostridial overgrowth, and recurrence of Clostridium difficile infection in mice Antimicrob Agents Chemother **57**, 689-696 10.1128/AAC.00877-12
11. Cosmetic Ingredient Review Expert, P. (2007) Final report on the safety assessment of Glycyrrhetinic Acid, Potassium Glycyrrhetinate, Disodium Succinoyl Glycyrrhetinate, Glyceryl Glycyrrhetinate, Glycyrrhetinyl Stearate, Stearyl Glycyrrhetinate, Glycyrrhizic Acid, Ammonium Glycyrrhizate, Dipotassium Glycyrrhizate, Disodium Glycyrrhizate, Trisodium Glycyrrhizate, Methyl Glycyrrhizate, and Potassium Glycyrrhizinate Int J Toxicol **26 Suppl 2**, 79-112 10.1080/10915810701351228
